# Supplementary material for: Socioeconomic deprivation and regional variation in Hodgkin’s lymphoma incidence in the UK: a population-based cohort study of 10 million individuals
Source: BMJ Open. 2019 Sep 20;9(9):e029228. doi: 10.1136/bmjopen-2019-029228 (PMC6756616; doi:10.1136/bmjopen-2019-029228)
Supplement: Supplementary data [file bmjopen-2019-029228supp001.pdf]

Supplementary Tables:

Supplementary Table 1: Read codes for Hodgkin's Lymphoma

B61..00	Hodgkin's disease
B61..11	Hodgkin lymphoma
B610.00	Hodgkin's paragranuloma
B610100	Hodgkin's paragranuloma of lymph nodes of head, face, neck
B610300	Hodgkin's paragranuloma of intra-abdominal lymph nodes
B611.00	Hodgkin's granuloma
B611100	Hodgkin's granuloma of lymph nodes of head, face and neck
B612.00	Hodgkin's sarcoma
B612400	Hodgkin's sarcoma of lymph nodes of axilla and upper limb
B613.00	Hodgkin's disease, lymphocytic-histiocytic predominance
B613000	Hodgkin's, lymphocytic-histiocytic predominance unspec site
B613100	Hodgkin's, lymphocytic-histiocytic pred of head, face, neck
B613200	Hodgkin's, lymphocytic-histiocytic pred intrathoracic nodes
B613300	Hodgkin's, lymphocytic-histiocytic pred intra-abdominal node
B613500	Hodgkin's, lymphocytic-histiocytic pred inguinal and leg
B613600	Hodgkin's, lymphocytic-histiocytic pred intrapelvic nodes
B613700	Hodgkin's, lymphocytic-histiocytic predominance of spleen
B613800	Hodgkin's, lymphocytic-histiocytic pred of multiple sites
B613z00	Hodgkin's, lymphocytic-histiocytic predominance NOS
B614.00	Hodgkin's disease, nodular sclerosis
B614000	Hodgkin's disease, nodular sclerosis of unspecified site
B614100	Hodgkin's nodular sclerosis of head, face and neck
B614200	Hodgkin's nodular sclerosis of intrathoracic lymph nodes
B614300	Hodgkin's nodular sclerosis of intra-abdominal lymph nodes
B614400	Hodgkin's nodular sclerosis of lymph nodes of axilla and arm
B614700	Hodgkin's disease, nodular sclerosis of spleen

B614800	Hodgkin's nodular sclerosis of lymph nodes of multiple sites
B614z00	Hodgkin's disease, nodular sclerosis NOS
B615.00	Hodgkin's disease, mixed cellularity
B615000	Hodgkin's disease, mixed cellularity of unspecified site
B615100	Hodgkin's mixed cellularity of lymph nodes head, face, neck
B615200	Hodgkin's mixed cellularity of intrathoracic lymph nodes
B615500	Hodgkin's mixed cellularity of lymph nodes inguinal and leg
B615z00	Hodgkin's disease, mixed cellularity NOS
B616.00	Hodgkin's disease, lymphocytic depletion
B616000	Hodgkin's lymphocytic depletion of unspecified site
B616400	Hodgkin's lymphocytic depletion lymph nodes axilla and arm
B616700	Hodgkin's disease, lymphocytic depletion of spleen
B616800	Hodgkin's lymphocytic depletion lymph nodes multiple sites
B616z00	Hodgkin's disease, lymphocytic depletion NOS
B617.00	Nodular lymphocyte predominant Hodgkin lymphoma
B618.00	Nodular sclerosis classical Hodgkin lymphoma
B619.00	Mixed cellularity classical Hodgkin lymphoma
B61B.00	Lymphocyte-rich classical Hodgkin lymphoma
B61C.00	Other classical Hodgkin lymphoma
B61z.00	Hodgkin's disease NOS
B61z.11	Hodgkin lymphoma NOS
B61z000	Hodgkin's disease NOS, unspecified site
B61z100	Hodgkin's disease NOS of lymph nodes of head, face and neck
B61z200	Hodgkin's disease NOS of intrathoracic lymph nodes
B61z300	Hodgkin's disease NOS of intra-abdominal lymph nodes
B61z400	Hodgkin's disease NOS of lymph nodes of axilla and arm
B61z500	Hodgkin's disease NOS of lymph nodes inguinal region and leg
B61z700	Hodgkin's disease NOS of spleen
B61z800	Hodgkin's disease NOS of lymph nodes of multiple sites

B61zz00	Hodgkin's disease NOS
BBj..00	[M]Hodgkin's disease
BBj0.00	[M]Hodgkin's disease NOS
BBj1.00	[M]Hodgkin's disease, lymphocytic predominance
BBj1000	[M]Hodgkin,s disease, lymphocytic predominance, diffuse
BBj1100	[M]Hodgkin,s disease, lymphocytic predominance, nodular
BBj2.00	[M]Hodgkin's disease, mixed cellularity
BBj4.00	[M]Hodgkin's disease,lymphocytic depletion,diffuse fibrosis
BBj6.00	[M]Hodgkin's disease, nodular sclerosis NOS
BBj6000	[M]Hodgkin,s disease, nodular sclerosis, lymphocytic predom
BBj6100	[M]Hodgkin,s disease, nodular sclerosis, mixed cellularity
BBj6200	[M]Hodgkin,s disease, nodular sclerosis, lymphocytic deplet
BBj7.00	[M]Hodgkin's disease, nodular sclerosis, cellular phase
BBj9.00	[M]Hodgkin's granuloma
BBjz.00	[M]Hodgkin's disease NOS
ByuD000	[X]Other Hodgkin's disease
ZV10711	[V]Personal history of Hodgkin's disease

Supplementary Table 2: ICD10 codes for Hodgkin’s Lymphoma

Code	Term
C81	Hodgkin lymphoma
C81.0	Nodular lymphocyte predominant Hodgkin lymphoma
C81.1	Nodular sclerosis (classical) Hodgkin lymphoma
C81.2	Mixed cellularity (classical) Hodgkin lymphoma
C81.3	Lymphocyte depleted (classical) Hodgkin lymphoma
C81.4	Lymphocyte-rich (classical) Hodgkin lymphoma
C81.7	Other (classical) Hodgkin lymphoma

C81.9	Hodgkin lymphoma unspecified

Supplementary Table 3: Age-specific incidence rates of Hodgkin's Lymphoma by sex. CI, confidence interval; ASR, age standardised rate

Age group (years)	Incidence Rate Overall	95% CI	Incidence Rate Males	95% CI	Incidence Rate Females	95% CI	Incidence Rate Ratio (males / females)
0-4	0.09	0.03–0.27	0.17	0.05–0.52	0.00	–	
5-9	0.52	0.35–0.77	0.50	0.29–0.89	0.54	0.31–0.95	0.94
10-14	1.48	1.17–1.88	1.63	1.19–2.23	1.32	0.92–1.90	1.23
15-19	3.40	2.89–4.00	3.09	2.45–3.90	3.75	3.00–4.70	0.82
20-24	3.83	3.29–4.47	3.53	2.83–4.41	4.16	3.36–5.16	0.85
25-29	3.55	3.05–4.12	3.37	2.71–4.19	3.73	3.02–4.59	0.90
30-34	2.72	2.31–3.20	3.49	2.85–4.27	1.94	1.48–2.55	1.80
35-39	3.18	2.76–3.67	3.76	3.13–4.53	2.59	2.06–3.24	1.46
40-44	2.48	2.11–2.91	3.14	2.57–3.83	1.80	1.38–2.35	1.74
45-49	2.44	2.07–2.87	3.01	2.45–3.70	1.85	1.41–2.41	1.63
50-54	2.87	2.46–3.35	3.53	2.89–4.29	2.20	1.70–2.83	1.61
55-59	3.38	2.91–3.92	3.63	2.96–4.44	3.12	2.50–3.89	1.16
60-64	3.90	3.37–4.51	5.04	4.20–6.05	2.77	2.17–3.54	1.82
65-69	4.51	3.90–5.22	5.15	4.23–6.26	3.92	3.15–4.87	1.31
70-74	5.58	4.83–6.43	6.47	5.33–7.86	4.80	3.89–5.92	1.35
75-79	5.18	4.40–6.10	5.80	4.58–7.32	4.71	3.75–5.91	1.23
80-84	5.06	4.17–6.15	6.49	4.93–8.54	4.15	3.15–5.46	1.56
85-89	4.91	3.79–6.37	4.67	2.94–7.41	5.03	3.68–6.89	0.93
90+	2.45	1.48–4.07	1.93	0.62–5.99	2.63	1.49–4.63	0.73
**ASR**	**3.10**	**2.98–3.22**	**3.51**	**3.32–3.70**	**2.72**	**2.56–2.88**	
