# Supplementary material for: Socioeconomic deprivation and regional variation in Hodgkin’s lymphoma incidence in the UK: a population-based cohort study of 10 million individuals
Source: BMJ Open. 2019 Sep 20;9(9):e029228. doi: 10.1136/bmjopen-2019-029228 (PMC6756616; doi:10.1136/bmjopen-2019-029228)
Supplement: Supplementary data [file bmjopen-2019-029228supp002.pdf]

**Supplementary Table 4:** Crude and age-standardised Hodgkin's Lymphoma incidence rates in the UK by sex, age group, deprivation and geographical region. PYAR, person years at risk; ASR, age standardised rate; CI, confidence interval; IMD, index of multiple deprivation; yrs, age group in years

| | Cases | PYAR | Incidence Rate per 100,000 PYAR | ASR per 100,000 PYAR | 95%CI |
|---|---|---|---|---|---|
| Male | 1331 | 39,039,332 | 3.41 | 3.51 | 3.32–3.70 |
| Female | 1071 | 39,529,340 | 2.71 | 2.72 | 2.56–2.88 |
| IMD 1 (least deprived) | 572 | 14,880,179 | 3.84 | 3.92 | 3.60–4.24 |
| IMD 2 | 500 | 14,627,384 | 3.42 | 3.49 | 3.18–3.79 |
| IMD 3 | 456 | 15,954,225 | 2.86 | 2.86 | 2.60–3.13 |
| IMD 4 | 472 | 16,850,996 | 2.80 | 2.82 | 2.57–3.07 |
| IMD 5 (most deprived) | 402 | 16,256,652 | 2.47 | 2.55 | 2.30–2.80 |
| North East England | 53 | 1,419,931 | 3.73 | 3.89 | 2.84–4.94 |
| Yorkshire/Humber | 105 | 2,938,253 | 3.57 | 3.64 | 2.94–4.33 |
| London | 299 | 8,287,047 | 3.61 | 3.82 | 3.38–4.25 |
| South East Coast | 284 | 7,564,683 | 3.75 | 3.79 | 3.35–4.23 |
| East of England | 213 | 7,012,254 | 3.04 | 3.09 | 2.68–3.51 |
| North West England | 275 | 9,159,626 | 3.00 | 3.06 | 2.70–3.42 |
| South West England | 197 | 6,598,934 | 2.99 | 2.95 | 2.54–3.36 |
| West Midlands | 198 | 7,054,011 | 2.81 | 2.84 | 2.44–3.24 |
| Wales | 202 | 7,155,514 | 2.82 | 2.81 | 2.42–3.20 |
| South Central England | 256 | 8,482,798 | 3.02 | 3.07 | 2.70–3.45 |
| East Midlands | 80 | 3,042,062 | 2.63 | 2.65 | 2.07–3.23 |
| Northern Ireland | 70 | 2,597,902 | 2.69 | 2.82 | 2.16–3.48 |
| Scotland | 170 | 7,256,423 | 2.34 | 2.35 | 2.00–2.70 |
| 0-4yrs | 3 | 3,507,576 | 0.09 | | |
| 5-9yrs | 24 | 4,620,605 | 0.52 | | |
| 10-14yrs | 68 | 4,590,771 | 1.48 | | |
| 15-19yrs | 147 | 4,321,501 | 3.40 | | |
| 20-24yrs | 162 | 4,226,413 | 3.83 | | |
| 25-29yrs | 170 | 4,794,244 | 3.55 | | |
| 30-34yrs | 147 | 5,402,587 | 2.72 | | |
| 35-39yrs | 187 | 5,877,364 | 3.18 | | |
| 40-44yrs | 151 | 6,089,433 | 2.48 | | |
| 45-49yrs | 145 | 5,943,893 | 2.44 | | |
| 50-54yrs | 159 | 5,541,281 | 2.87 | | |
| 55-59yrs | 172 | 5,094,300 | 3.38 | | |
| 60-64yrs | 179 | 4,592,514 | 3.90 | | |
| 65-69yrs | 181 | 4,009,358 | 4.51 | | |
| 70-74yrs | 189 | 3,390,115 | 5.58 | | |
| 75-79yrs | 144 | 2,779,666 | 5.18 | | |
| 80-84yrs | 102 | 2,015,256 | 5.06 | | |
| 85-89yrs | 57 | 1,160,678 | 4.91 | | |
| 90+yrs | 15 | 611,886 | 2.45 | | |
| **Overall ASR** | | | | **3.10** | **2.98–3.22** |
