# Supplementary material for: Socioeconomic deprivation and regional variation in Hodgkin’s lymphoma incidence in the UK: a population-based cohort study of 10 million individuals
Source: BMJ Open. 2019 Sep 20;9(9):e029228. doi: 10.1136/bmjopen-2019-029228 (PMC6756616; doi:10.1136/bmjopen-2019-029228)
Supplement: Author's manuscript [file bmjopen-2019-029228.draft_revisions.pdf]

BMJ Open

BMJ Open is committed to open peer review. As part of this commitment we make the peer review history of every article we publish publicly available.

When an article is published we post the peer reviewers' comments and the authors' responses online. We also post the versions of the paper that were used during peer review. These are the versions that the peer review comments apply to.

The versions of the paper that follow are the versions that were submitted during the peer review process. They are not the versions of record or the final published versions. They should not be cited or distributed as the published version of this manuscript.

BMJ Open is an open access journal and the full, final, typeset and author-corrected version of record of the manuscript is available on our site with no access controls, subscription charges or pay-per-view fees (http://bmjopen.bmj.com).

If you have any questions on BMJ Open's open peer review process please email info.bmjopen@bmj.com

BMJ Open

Socioeconomic deprivation and regional variation in Hodgkin's lymphoma incidence in the UK: A population-based cohort study of 10 million individuals

Journal:	*BMJ Open*
Manuscript ID	bmjopen-2019-029228
Article Type:	Research
Date Submitted by the Author:	17-Jan-2019
Complete List of Authors:	Rafiq, Meena; University College London, Institute of Health Informatics<br>Hayward, Andrew; University College London, Institute of Epidemiology and Health Care<br>Warren-Gash, Charlotte; London School of Hygiene and Tropical Medicine, Non-communicable disease epidemiology<br>Denaxas, S; University College London, Institute of Health Informatics<br>Gonzalez-Izquierdo, Arturo; University College London, Institute of Health Informatics<br>Lyratzopoulos, Georgios ; University College London, Department of Epidemiology & Public Health, Health Behaviour Research Centre<br>Thomas, Sara; London School of Hygiene & Tropical Medicine, Faculty of Epidemiology & Population Health
Keywords:	Epidemiology < ONCOLOGY, Lymphoma < ONCOLOGY, Lymphoma < HAEMATOLOGY

SCHOLARONE™
Manuscripts

Socioeconomic deprivation and regional variation in Hodgkin's lymphoma incidence in the UK: A population-based cohort study of 10 million individuals

Meena Rafiq[1], Andrew Hayward[2], Charlotte Warren-Gash[3], Spiros Denaxas[1], Arturo Gonzalez-Izquierdo[1], Georgios Lyratzopoulos[2] and Sara Thomas[3]

[1]Institute of Health Informatics, UCL, London, UK

[2]UCL institute of Epidemiology and Health Care, UCL, London, UK

[3]London School of Hygiene and Tropical Medicine, London, UK

For peer review only

Key Words: Hodgkin's lymphoma; epidemiology; UK; deprivation; regional variation

Corresponding Author: Dr Meena Rafiq**,** Institute of Health Informatics, University College London, 222 Euston Road, London, NW1 2DA, UK

Email: Meena.rafiq@ucl.ac.uk

Tel: +44 20 3549 5321

Word count:

Abstract:	284
Main Text:	3177
Number of Figures:	3
Number of Tables:	1

Funding:

Dr Meena Rafiq and the work presented in this paper are funded by a National Institute for Health Research (NIHR) in-practice clinical fellowship (IPF-2017-11-011). This article presents independent research funded by the NIHR. The views expressed are those of the author(s) and not necessarily those of the NHS, the NIHR

or the Department of Health. GL is supported by Cancer Research UK Advanced Clinician Scientist Fellowship (C18081/A18180).

Conflict of interest: The authors declare no potential conflicts of interest.

Author Contributions: MR, AH, CWG and ST contributed to the conception and design of the study, planning of analyses, interpretation of results and writing the manuscript. MR, SD and AGI contributed to the planning of the analyses, extracting the data and performing the statistical analyses. GL contributed to study design and interpretation of results. All authors have read and approved the final manuscript.

Data sharing statement CPRD data on which the study was based is held securely by University College London under the CPRD data access licence (https://www.cprd.com/dataAccess/).

For peer review only

For peer review only - http://bmjopen.bmj.com/site/about/guidelines.xhtml

Abstract:

Objectives:

Hodgkin's Lymphoma (HL) is the commonest cancer in teenagers and young adults. This population-based cohort study conducted over a 25-year period in the UK investigates variation in HL incidence by age, sex, region and deprivation to identify trends and high-risk populations for HL development.

Setting:

Electronic primary care records linked to Hospital Episode Statistics and Index of Multiple Deprivation data were used.

Participants:

Data on 10 million UK individuals from 1992–2016 were analysed.

Outcome measures:

Poisson models were used to explore differences in HL incidence by age, sex, region and deprivation by estimating incidence rate ratios (IRR). Age-specific HL incidence rates by sex and directly age-standardised incidence rates by region and deprivation group were calculated.

Results:

2,402 new cases of HL were identified over 78,569,436 person years. There was significant variation in HL incidence by deprivation group. Individuals living in the most affluent areas had HL incidence 60% higher than those living in the most deprived (IRR 1.60, 95% confidence interval 1.40–1.83), with strong evidence of a marked linear trend towards increasing HL incidence with decreasing deprivation (p=<0.001). There was significant regional variation in HL incidence across the UK,

For peer review only

which persisted after adjusting for age, sex and deprivation (IRR 0.80–1.42, p=<0.001).

Conclusions:

This study identified high-risk regions for HL development in the UK and observed a trend towards higher incidence of HL in individuals living in less deprived areas. Consistent with findings from other immune-mediated diseases, this study supports that an affluent childhood environment with fewer immune challenges affects immune maturation in early life, thereby predisposing to development of immune-related neoplasms. Understanding the mechanisms behind this immune dysfunction could inform prevention, detection and treatment of HL and other immune diseases.

Article Summary:

Strengths and limitations of this study

- Our population-based data covered a large representative sample of over 10 million individuals in the UK over a 25-year period with 78 million years of follow up.
- We used UK primary care electronic health records linked to secondary care data and Index of Multiple Deprivation data to improve capture of Hodgkin's lymphoma diagnoses and allow analysis of geographical and deprivation based trends.
- Data from the Clinical Practice Research Datalink (CPRD) used in this study have been demonstrated to be generalisable to the UK population across a number of demographics.
- Data in this study were not linked to the National Cancer Register (NCR), which is a potential limitation; however lymphoma diagnosis in CPRD has

For peer review only

been validated in previous studies and shown to have high concordance with the NCR.

- This is a cohort study of a representative sample of the UK population and not the whole UK population.

For peer review only

For peer review only - http://bmjopen.bmj.com/site/about/guidelines.xhtml

Introduction:

Hodgkin's Lymphoma (HL) is the commonest cancer in teenagers and young adults worldwide (1, 2). In the UK 2,100 new cases of HL are diagnosed each year, but little is known about the distribution of these cases in the UK population or if there are any high-risk groups. International studies have identified that HL incidence varies considerably between countries, with higher rates observed in high-income countries (3-7). This pattern is also seen within countries, with US studies showing higher rates in more affluent regions and geographical variation in HL incidence between different states (8). Few UK studies have investigated HL incidence patterns by socioeconomic deprivation (9-12) and region (12-14) and the results have been conflicting and inconclusive. Additionally, to our knowledge there have been no recent studies investigating patterns of HL incidence in the UK population since 2010.

Understanding how HL incidence varies between different geographical regions in the UK and identifying high-risk populations may provide clues to the underlying aetiology of the disease and inform future research directions. We aimed to conduct a population-based cohort study of 10 million individuals over a 25-year period using linked primary and secondary care electronic health records to investigate variation in HL incidence within the UK by age, sex, geographic region and deprivation.

Materials and methods:

Data sources and study population

For peer review only - http://bmjopen.bmj.com/site/about/guidelines.xhtml

For peer review only

Data were obtained from the UK Clinical Practice Research Datalink (CPRD), linked to Hospital Episode Statistic (HES) inpatient data and Index of Multiple Deprivation (IMD) data. CPRD is an electronic health record database containing prospectively collected pseudo-anonymised data from UK primary care consultations. It is the largest source of longitudinal primary care data, holding information on 22 million patients representing approximately 9% of the UK population (in 2013) (15). This database has been shown to be largely representative of the UK population across a number of demographics including age, sex and ethnicity (15). Data are available from 1987 onwards when CPRD was first established. Practices contributing to CPRD are regularly audited to ensure high data quality and that 95% of prescribing and morbidity events are captured before practices are declared 'up-to-standard' (UTS) for research purposes (16). HES data provide additional information from hospital attendances in England. IMD scores represent a composite ecological (small-area based) measure of the socioeconomic status of a patient, based on the income, employment, disability, educational attainment and other attributes of the LSOA (Local Super Output Area) of a patients' residence. The latter typically comprise populations between 1,000 and 3,000 residents. All patients had an aggregate IMD score pertaining to the LSOA of their own residence or that of their general practice.

The study population comprised patients actively registered with a CPRD practice between January 1992 and December 2016 who did not have a pre-existing diagnosis of HL. In accordance with previous studies, eligible follow-up time in days for each patient was commenced from one year after the patient registered with the practice (to avoid capturing past diagnoses recorded retrospectively in the few months after new patient registration) (17), or from when CPRD classified the GP practice to be 'up-to-standard' (UTS) if this occurred later. Active follow-up ended

For peer review only - http://bmjopen.bmj.com/site/about/guidelines.xhtml
For peer review only

when a patient received a diagnosis of HL, died, left a CPRD practice or at the last data collection date for participating practices, whichever occurred earlier.

Classification of outcome and exposure

Data were obtained on HL diagnoses coded using Read codes (in CPRD) or the International Classifications of Diseases, 10th Revision codes (ICD-10, in HES) (Supplementary Table S1 and S2); age and date of diagnosis; area of residence by Strategic Health Authority (SHA) region; deprivation using IMD quintiles; date of birth and sex.

Statistical analysis

For each new case of HL, the year and age at diagnosis were determined and the patient was counted as an incident case for that calendar year and age group. The duration of active follow-up in CPRD for each individual in the study population was then calculated and used to calculate the total person years at risk (PYAR) and to estimate crude HL incidence rates per 100,000 PYAR.

Age-specific HL incidence rates were calculated in 5-year age bands, first for persons and then stratified by sex. Age-standardised incidence rates were estimated by the direct method using the European standard population for each region and deprivation quintile. Poisson regression was used to model HL incidence rate ratios (IRRs) for region, deprivation, age and sex independently before adjusting for other variables. East of England was used as the reference category for region, as the region with age-standardised incidence estimate that was closest to the national average (18). Deprivation was initially included as a categorical variable in the regression analysis to calculate IRRs and then subsequently we assessed for a

For peer review only

For peer review only - http://bmjopen.bmj.com/site/about/guidelines.xhtml

linear trend by deprivation quintile, first by estimating the linear effect of deprivation using likelihood ratio tests, and then investigating departure from linearity by comparing models in which deprivation was added as a non-linear vs. a linear term. Additionally, incidence rates by deprivation were examined to see if any variation persisted after adjusting for trends in region, and vice versa to see if trends in region were observed after adjusting for deprivation as a categorical variable. Adjusted models were also adjusted for age and sex.

HL has a bimodal age-specific incidence pattern with the first peak occurring between 15-34 years and a second peak between 70-84 years (18). Previous studies have suggested HL in individuals aged <50 and >50 is likely to have different aetiological factors. We therefore performed pre-specified subgroup analyses by sex (male vs. female) and age (≤50 vs. >50 years). We additionally examined interactions between exposure variables in the final model, particularly given potential variation in risk of HL by age and sex (18) i.e. age group by sex, age group by deprivation, age group by region, sex by deprivation and sex by region. Deprivation group was treated as a categorical variable in interaction terms. Analyses were performed using Stata (version 15; StataCorp, College Station, TX, USA). The protocol for this project was approved by the LSHTM Ethics Committee (ref:11182) and the ISAC for MHRA Database Research (protocol number:16_237). Generic ethical approval for observational studies conducted using anonymised CPRD data with approval from ISAC has been granted from a National Research Ethics Service Committee (NRESC).

Results:

For peer review only - http://bmjopen.bmj.com/site/about/guidelines.xhtml

There were 2,402 new diagnoses of HL identified over the 25-year study period (78,569,436 person years of follow-up) with an overall HL incidence of 3.06 cases per 100,000 PYAR (95% confidence interval (CI) 2.94–3.18). Age-specific HL incidence showed a bimodal distribution with an initial peak at ages 20–24 years followed by a second peak at ages 70–74 years characteristic of HL incidence in high-income countries (Figure 1, Supplementary Table S3 and S4). Incidence was higher in older adults compared to those aged ≤50 (4.12 cases per 100,000 PYAR, 95%CI 3.89–4.36 vs. 2.46 cases per 100,000 PYAR, 95%CI 2.32–2.59) and was higher in males than in females in all age groups (with an overall IRR for males vs. females of 1.26, 95%CI 1.16-1.36 and age-specific IRRs ranging from 1.16–1.82) except for 15–29 years when incidence in females exceeded that of males (age-specific IRRs 0.82-0.90) and at the extremes of the age range where the number of cases were small (Figure 1, Supplementary Table S3). Of the 2,402 incident cases of HL, 52.8% were identified in HES (407 not in CPRD), 83.1% in CPRD (1,133 not in HES) and 35.9% were identified in both.

Regional variation

Age-standardised incidence rates showed regional variation in HL incidence across the UK with the North East of England having the highest rates (3.89 cases per 100,000 PYAR) and Scotland having the lowest (2.35 cases per 100,000 PYAR) (Figure 2, Supplementary Table S4).

Multivariable Poisson regression revealed strong evidence for an association between geographical region and HL incidence (Table 1), which persisted after adjusting for deprivation, age and sex (p=<0.001). HL incidence rates were significantly higher than the reference population rate (East of England) for individuals living in North East England, Yorkshire and Humber, London and the

For peer review only

South East Coast and significantly lower for those living in Scotland, after adjusting for other variables. Subgroup analysis showed that regional variation in HL incidence was observed in both males and females, but was limited to individuals aged over 50 years, with no evidence for an association between region and HL incidence demonstrated in the younger age group (p=0.23).

Socioeconomic deprivation

There was strong evidence for an association between HL incidence and deprivation (p=<0.001), with age-standardised incidence being highest in the most affluent population groups and lowest in the most deprived (3.92 cases per 100,000 PYAR vs. 2.55 cases per 100,000 PYAR, Supplementary Table S4). Poisson regression showed the least deprived individuals in the population had HL incidence rates over 50% higher than the most deprived after adjusting for other factors (IRR = 1.55 and 1.63 for individuals aged <50 vs. ≥50 respectively, Table 1). The strong evidence of a marked linear trend towards lower rates of HL incidence with increasing deprivation persisted after adjusting for region and was observed across both sexes and when analysing young and old adults separately (Figure 3, Table 1).

Interaction analysis

There was no evidence that regional differences in HL incidence varied by age or sex ($P_{interaction}$= 0.40 and 1.00 respectively). Additionally, there was no evidence that the association between deprivation and HL risk varied by age or sex ($P_{interaction}$= 0.57 and 0.39 respectively). The characteristic bimodal age-specific HL incidence pattern was observed in both males and females, and the association between age and HL incidence did not vary by sex ($P_{interaction}$= 0.16).

For peer review only - http://bmjopen.bmj.com/site/about/guidelines.xhtml
For peer review only

Discussion:

This is the largest study to date investigating variability in HL incidence by region and deprivation. It uses comprehensive linked electronic primary care records over a 25-year period in a representative cohort of the UK population. We found strong evidence that reducing deprivation is associated with a higher incidence of HL, and this is the first study to demonstrate that this trend is observed in older adults as well as in children and young adults. There was considerable variation in HL incidence by UK geographical region, and these differences persisted even when sex, age and deprivation were taken into account.

Comparison with the literature

The bimodal age-specific HL incidence pattern described in this study is consistent with findings from other high-income countries, including a single previous study in the UK (3, 5-7, 18-20). Higher incidence of HL in males except between ages 15-29 was also observed in a previous UK study, which found an isolated higher incidence in females aged 15-24 (18). When looking at the association with deprivation previous studies have shown heterogeneous outcomes. One previous UK study found that HL incidence in males between 2006-2010 was greater in more deprived areas, with no association observed between deprivation and HL at any other point in the study in either sex (1996-2010) (9). Another study investigating the distribution of childhood cancers in the UK between 1969-1993 found HL incidence in children aged 0-9 was greater in more deprived areas (11). In contrast, two previous studies conducted in parts of England and Wales found a marked linear trend towards increasing HL incidence with higher socioeconomic status in individuals aged 0-24 (10, 12). Our findings were concordant with these studies and demonstrated increased HL incidence in more affluent individuals in a larger population of 10

For peer review only - http://bmjopen.bmj.com/site/about/guidelines.xhtml

million people over a longer time period, broader geographic area and in a wider age range. The observation of this trend in older adults in this study is a new finding, which to our knowledge has not been previously reported. With regards to regional variation the previous literature has also been conflicting. Quinn et al. and McKinney et al. reported no clear geographical variation in HL incidence rates within the UK population (13, 14). Alston et al. reported conflicting findings with strong evidence that HL incidence varied by UK region and observed elevated incidence of HL in London and the South East of England in individuals aged 0-24 years (12). These findings were in line with those observed in this long-term population study, which demonstrated significant regional variation in HL incidence across the UK with elevated rates in London and the South East. Regional variations were present in both males and females, but interestingly were limited to older adults, although this may have been due to small numbers rather than a true lack of an effect in younger adults.

Strengths and limitations

The main strengths of our study are that it is a large population-based study of more than 10 million individuals and has a long period of case ascertainment. HL is a relatively rare disease and the size of this study gives it the power to detect smaller effect sizes and associations that could be missed in smaller studies. Additionally, it allows examination of inter-relationships between variables to see the extent to which trends are influenced by other factors and enables subgroup analysis by age and sex. This is particularly important due to the growing evidence for two potentially separate aetiological pathways underlying HL incidence in young and older adults, and therefore the need for them to be analysed independently. A further advantage of this study was the use of CPRD data with regional information, linked to HES and deprivation data. CPRD has wide national coverage and has been demonstrated to

For peer review only - http://bmjopen.bmj.com/site/about/guidelines.xhtml

be representative of the UK population across a number of demographics making the results generalisable to the UK population (15).

The main limitation of this study is the that it did not have access to linked data from the UK National Cancer Registry (NCR), which is the gold standard for identification of incident cases of HL in the UK population. This could result in potential misclassification of cases as controls in this study and subsequent underestimation of effect estimates. Previous concordance studies have demonstrated that HL diagnoses have high validity in CPRD when compared to the NCR (positive predictive value for lymphoma 89.6%, sensitivity 97.3%) and this effect is therefore likely to be minimal (21). Additionally, CPRD has established use in cancer epidemiology in the literature (21-24) and use of HES-linked data further improved validity of HL diagnosis by supplementing GP records with hospital data to improve capture of diagnoses. Another limitation is the use of routinely collected data with potential misclassification of an individual's deprivation group. Deprivation was determined using IMD which is based on the postcode of the patients residence or registered GP practice and not on individual-level characteristics. As there may be variation in deprivation within a postcode, especially in highly diverse inner-city areas, this could result in non-differential misclassification of deprivation and underestimation of any effects. Additionally, the deprivation quintile and region captured from the dataset and used in the study may not represent childhood deprivation groups and region of residence, which may be more appropriate if early-life exposures are involved in the etiology of HL. The earliest available linked IMD scores (2004 for patient level and 2009 for practice-level) were used in this study to estimate deprivation. This assumes both that an individuals IMD status remains stable throughout their life, and that the IMD quintile of a postcode remains stable over time. Both of these assumptions may not be true as individuals can move between deprivation quintiles and areas may undergo gentrification over time.

For peer review only

For peer review only - http://bmjopen.bmj.com/site/about/guidelines.xhtml

Population movement also means an individuals childhood residence may differ from their current regional residence, which could dilute any regional variation observed in HL incidence.

Implications

The bimodal incidence pattern and differences in regional variation between younger versus older adults supports the hypothesis that there may be different aetiological pathways involved in the development of HL in these age groups (3, 4, 19). This is further supported by evidence from previous studies for different distributions of the histological subtypes of HL between the two age groups (5, 7, 8, 25-28). Consideration should be given to investigating HL aetiology separately in these age groups in future studies to identify potential different contributory factors that could be masked when analysing the population as a whole. Additionally, the existence of potentially different pathophysiology could have important implications for targeting and response to treatment regimens and in disease monitoring and detection. The peak in disease incidence in young adult females is characteristic of a number of immune-related conditions, including multiple sclerosis, rheumatoid arthritis and lupus (29-31). Similarities between incidence patterns for these diseases could suggest a common predisposing factor in early life that interferes with immune regulation and promotes development of immune-related diseases in young adults.

The trend towards increased HL incidence with increased affluence was replicated across three separate UK databases and is consistent with findings from US studies (8, 10, 12). Concordance between these findings add further support for this being a true association. This trend has been previously well established in ecological studies making comparisons between countries with very different levels of deprivation (3-7). Within country differences in deprivation tend to be much smaller

For peer review only

than those seen between countries. Our results could suggest that even small increases in community deprivation levels may elevate an individual's risk of developing HL. A proposed explanation for this association is that children in affluent households with less overcrowding and cleaner childhood environments consequently have delayed exposure to infectious agents and fewer immune challenges in early-life to stimulate immune development and regulation (32-37). This predisposes them to develop immune related conditions(38-40). Observation of this trend in older adults is less likely to be explained by childhood exposures. HL aetiology could be multifactorial with childhood exposures predisposing individuals, but in the absence of other promoting factors in early-life, onset of HL is delayed until later adulthood. This should be further explored in future studies to identify contributory factors underlying the association in older adults.

Regional variation in HL incidence was observed after adjusting for deprivation differences in older adults. This indicates that other factors that vary geographically in these regions are contributing to increased HL incidence in this age group. Geographical clustering of HL cases has been previously reported in both the UK and USA (8, 12, 41-44), which could support the role for an environmental factor underlying increased rates in these regions. Other possible contributory factors include regional differences in ethnicity and clustering of predisposing or protective genotypes. Further studies are required to investigate the role of these different factors in regional variation in UK HL incidence.

Conclusion

In conclusion, this study of over 10 million individuals based on nationwide primary care data found strong evidence for regional variation in HL incidence across the UK that cannot be explained by geographical differences in deprivation. More affluent

For peer review only - http://bmjopen.bmj.com/site/about/guidelines.xhtml

individuals within the UK population have a significantly higher risk of developing HL in both younger and older adults. This trend has been observed for other immune-mediated diseases. It adds to the growing evidence that an affluent childhood environment with fewer immune challenges interferes with the maturation of the immune system and predisposes to development of immune-related conditions. Further understanding the responsible pathophysiological mechanisms could inform prevention, detection and treatment of HL and other immune conditions.

For peer review only

For peer review only - http://bmjopen.bmj.com/site/about/guidelines.xhtml

References:

1. Bleyer WA. Cancer in older adolescents and young adults: epidemiology, diagnosis, treatment, survival, and importance of clinical trials. Med Pediatr Oncol. 2002;38(1):1-10.

2. Alarcon PA. Pediatric Hodgkin Lymphoma http://emedicine.medscape.com/article/987101-overview - a5: Medscape, medicine; 2017 [

3. MACMAHON B. Epidemiological evidence of the nature of Hodgkin's disease. Cancer. 1957;10(5):1045-54.

4. MacMahon B. Epidemiology of Hodgkin's disease. Cancer Res. 1966;26(6):1189-201.

5. Correa P, O'Conor GT. Epidemiologic patterns of Hodgkin's disease. Int J Cancer. 1971;8(2):192-201.

6. Harris NL. The many faces of Hodgkin's disease around the world: what have we learned from its pathology? Ann Oncol. 1998;9 Suppl 5:S45-56.

7. Parkin D, Whelan S, Ferlay J, Teppo L, Thomas D. Cancer incidence in five continents, Vol. VIII, International Agency for Research on Cancer.(IARC Scientific Publication 155). Lyon, France: IARC Press; 2002.

8. Glaser SL. Regional variation in Hodgkin's disease incidence by histologic subtype in the US. Cancer. 1987;60(11):2841-7.

9. Network NCI. Cancer by Deprivation in England. Incidence, 1996-2010. Mortality, 1997-2011. 2014.

10. Alexander FE, Ricketts TJ, McKinney PA, Cartwright RA. Community lifestyle characteristics and incidence of Hodgkin's disease in young people. Int J Cancer. 1991;48(1):10-4.

11. Elliott A. Committee on Medical Aspects of Radiation in the Environment (COMARE). 11th report. The distribution of childhood leukaemia and other childhood cancers in Great Britain 1969–1993. . 2006.

12. Alston RD, Rowan S, Eden TO, Moran A, Birch JM. Cancer incidence patterns by region and socioeconomic deprivation in teenagers and young adults in England. Br J Cancer. 2007;96(11):1760-6.

13. Quinn M, Wood H, Cooper N, Rowan S. Cancer atlas of the United Kingdom and Ireland 1991–2000. Studies on medical and population subjects. 2005;68.

14. McKinney PA, Alexander FE, Ricketts TJ, Williams J, Cartwright RA. A specialist leukaemia/lymphoma registry in the UK. Part 1: Incidence and geographical distribution of Hodgkin's disease. Leukaemia Research Fund Data Collection Study Group. Br J Cancer. 1989;60(6):942-7.

15. Herrett E, Gallagher AM, Bhaskaran K, Forbes H, Mathur R, van Staa T, et al. Data Resource Profile: Clinical Practice Research Datalink (CPRD). Int J Epidemiol. 2015;44(3):827-36.

16. Digital N. Hospital Episode Statistics http://content.digital.nhs.uk/hes2017 [

17. Lewis JD, Bilker WB, Weinstein RB, Strom BL. The relationship between time since registration and measured incidence rates in the General Practice Research Database. Pharmacoepidemiol Drug Saf. 2005;14(7):443-51.

18. Cancer Research UK. Hodgkin lymphoma incidence statistics May 2018 [Available from: http://www.cancerresearchuk.org/health-professional/cancer-statistics/statistics-by-cancer-type/hodgkin-lymphoma - heading-Zero.

For peer review only

For peer review only - http://bmjopen.bmj.com/site/about/guidelines.xhtml

19. Hjalgrim H. On the aetiology of Hodgkin lymphoma. Dan Med J. 2012;59(7):B4485.
20. Clemmesen J. Statistical studies in the aetiology of malignant neoplasms. Acta Pathol Microbiol Scand Suppl. 1974;Suppl 247:1-266.
21. Boggon R, van Staa TP, Chapman M, Gallagher AM, Hammad TA, Richards MA. Cancer recording and mortality in the General Practice Research Database and linked cancer registries. Pharmacoepidemiol Drug Saf. 2013;22(2):168-75.
22. Dregan A, Moller H, Murray-Thomas T, Gulliford MC. Validity of cancer diagnosis in a primary care database compared with linked cancer registrations in England. Population-based cohort study. Cancer Epidemiol. 2012;36(5):425-9.
23. Arhi CS, Bottle A, Burns EM, Clarke JM, Aylin P, Ziprin P, et al. Comparison of cancer diagnosis recording between the Clinical Practice Research Datalink, Cancer Registry and Hospital Episodes Statistics. Cancer Epidemiol. 2018;57:148-57.
24. Margulis AV, Fortuny J, Kaye JA, Calingaert B, Reynolds M, Plana E, et al. Validation of Cancer Cases Using Primary Care, Cancer Registry, and Hospitalization Data in the United Kingdom. Epidemiology (Cambridge, Mass). 2018;29(2):308.
25. Franssila KO, Heiskala MK, Heiskala HJ. Epidemiology and histopathology of Hodgkin's disease in Finland. Cancer. 1977;39(3):1280-8.
26. Newell GR, Cole SR, Miettinen OS, MacMahon B. Age differences in the histology of Hodgkin's disease. Journal of the National Cancer Institute. 1970;45(2):311-7.
27. Henderson BE, Dworsky R, Pike MC, Baptista J, Menck H, Preston-Martin S, et al. Risk factors for nodular sclerosis and other types of Hodgkin's disease. Cancer Res. 1979;39(11):4507-11.
28. Cozen W, Katz J, Mack TM. Risk patterns of Hodgkin's disease in Los Angeles vary by cell type. Cancer Epidemiol Biomarkers Prev. 1992;1(4):261-8.
29. Whitacre CC, Reingold SC, O'Looney PA. A gender gap in autoimmunity. Science. 1999;283(5406):1277-8.
30. Jacobson DL, Gange SJ, Rose NR, Graham NM. Epidemiology and estimated population burden of selected autoimmune diseases in the United States. Clin Immunol Immunopathol. 1997;84(3):223-43.
31. Beeson PB. Age and sex associations of 40 autoimmune diseases. Am J Med. 1994;96(5):457-62.
32. Glaser SL, Lin RJ, Stewart SL, Ambinder RF, Jarrett RF, Brousset P, et al. Epstein-Barr virus-associated Hodgkin's disease: epidemiologic characteristics in international data. Int J Cancer. 1997;70(4):375-82.
33. Chang ET, Zheng T, Weir EG, Borowitz M, Mann RB, Spiegelman D, et al. Childhood social environment and Hodgkin's lymphoma: new findings from a population-based case-control study. Cancer Epidemiol Biomarkers Prev. 2004;13(8):1361-70.
34. Glaser SL, Clarke CA, Nugent RA, Stearns CB, Dorfman RF. Social class and risk of Hodgkin's disease in young-adult women in 1988-94. Int J Cancer. 2002;98(1):110-7.
35. Gutensohn N, Cole P. Epidemiology of hodgkin's disease in the young. Int J Cancer. 1977;19(5):595-604.
36. Mueller N. Hodgkin's disease. In: Schottendfeld D, Fraumeni JF Jr, eds. Cancer epidemiology and prevention. New York: Oxford University Press; 1996.

For peer review only

For peer review only - http://bmjopen.bmj.com/site/about/guidelines.xhtml

37. Mueller N, Grufferman S. The epidemiology of Hodgkin's disease. In: Mauch PM, Armitage JO, Diehl V, et al, eds. Hodgkin's disease. Philadelphia: Lippincott Williams & Wilkins; 1999.
38. Greaves M. Darwinian medicine: a case for cancer. Nature Reviews Cancer. 2007;7(3):213.
39. Greaves M. A causal mechanism for childhood acute lymphoblastic leukaemia. Nature Reviews Cancer. 2018:1.
40. Wills-Karp M, Santeliz J, Karp CL. The germless theory of allergic disease: revisiting the hygiene hypothesis. Nature Reviews Immunology. 2001;1(1):69.
41. Barnes N, Cartwright RA, O'Brien C, Roberts B, Richards ID, Bird CC. Spatial patterns in electoral wards with high lymphoma incidence in Yorkshire health region. Br J Cancer. 1987;56(2):169-72.
42. Cartwright RA, Watkins G. Epidemiology of Hodgkin's disease: a review. Hematol Oncol. 2004;22(1):11-26.
43. Smith PG. Current assessment of "case clustering" of lymphomas and leukemias. Cancer. 1978;42(2 Suppl):1026-4.
44. Alexander FE, Williams J, McKinney PA, Ricketts TJ, Cartwright RA. A specialist leukaemia/lymphoma registry in the UK. Part 2: Clustering of Hodgkin's disease. Br J Cancer. 1989;60(6):948-52.

For peer review only

Table 1: Hodgkin's lymphoma risk by sex, socioeconomic status and geographical region

	Adjusted IRR (95%CI)¥				
Risk factors	Study Population	≤50 years	>50 years	Males	Females
Sex					
Male	1.30 (1.20-1.41)	1.23 (1.10-1.38)	1.38 (1.23-1.55)		
p value	<0.001	<0.001	<0.001		
Region					
East of England	*ref*	*ref*	*ref*	*ref*	*ref*
North East England	1.42 (1.05-1.93)	0.82 (0.48-1.40)	2.05 (1.40-3.01)	1.49 (1.00-2.24)	1.34 (0.84-2.13)
Yorkshire/Humber	1.32 (1.04-1.68)	1.11 (0.78-1.58)	1.55 (1.12-2.13)	1.48 (1.08-2.01)	1.15 (0.79-1.66)
London	1.29 (1.08-1.54)	1.15 (0.90-1.48)	1.45 (1.13-1.87)	1.25 (0.99-1.59)	1.33 (1.02-1.73)
South East Coast	1.23 (1.03-1.48)	1.24 (0.96-1.59)	1.24 (0.97-1.59)	1.19 (0.94-1.51)	1.29 (0.99-1.69)
North West England	1.07 (0.89-1.28)	1.05 (0.82-1.36)	1.07 (0.83-1.39)	1.13 (0.89-1.43)	0.99 (0.75-1.31)
South West England	1.06 (0.87-1.29)	1.08 (0.82-1.43)	1.04 (0.78-1.37)	1.05 0.81-1.36)	1.07 (0.80-1.43)
West Midlands	1.00 (0.82-1.21)	0.90 (0.68-1.20)	1.10 (0.83-1.44)	0.96 (0.74-1.26)	1.04 (0.78-1.40)
Wales	0.97 (0.80-1.18)	1.08 (0.83-1.42)	0.87 (0.65-1.15)	0.96 (0.74-1.25)	0.98 (0.73-1.32)
South Central England	0.96 (0.80-1.15)	0.96 (0.75-1.24)	0.95 (0.73-1.23)	0.87 (0.68-1.11)	1.08 (0.82-1.42)
East Midlands	0.95 (0.73-1.23)	1.10 (0.78-1.54)	0.79 (0.53-1.17)	0.94 (0.67-1.34)	0.96 (0.65-1.42)
Northern Ireland	0.90 (0.68-1.17)	0.78 (0.53-1.15)	1.02 (0.70-1.48)	0.90 (0.63-1.29)	0.90 (0.60-1.06)
Scotland	0.80 (0.66-0.98)	0.89 (0.68-1.17)	0.71 (0.53-0.96)	0.82 (0.63-1.08)	0.78 (0.57-1.06)
p value	<0.001	0.23	<0.001	0.002	0.03
IMD quintile					
5 (most deprived)	*ref*	*ref*	*ref*	*ref*	*ref*
4	1.10 (0.96-1.26)	1.20 (1.00-1.45)	1.01 (0.83-1.23)	1.11 (0.92-1.34)	1.09 (0.89-1.33)
3	1.15 (1.00-1.32)	1.12 (0.92-1.36)	1.18 (0.97-1.44)	1.21 (1.00-1.47)	1.08 (0.88-1.33)
2	1.35 (1.18-1.55)	1.37 (1.13-1.66)	1.33 (1.10-1.62)	1.45 (1.21-1.75)	1.25 (1.02-1.52)
1 (least deprived)	1.60 (1.40-1.83)	1.55 (1.29-1.88)	1.63 (1.35-1.97)	1.87 (1.57-2.24)	1.31 (1.07-1.61)
p value	<0.001*	<0.001*	<0.001*	<0.001*	0.003*

Adjusted IRR, ¥incidence rate ratio adjusted for age, sex, region and IMD quintile; IMD, Index of Multiple Deprivation; CI, confidence interval; p, p value from likelihood-ratio test; * p value from test for linear trend; ref, reference group (East of England used as the reference category as the region with age-standardised incidence estimate that was closest to the national average)

For peer review only

Figure Legends:

Figure 1: Age-specific Hodgkin's Lymphoma incidence in the study population (cohort of UK population): overall (left panel) and by sex (right panel), with 95% confidence interval bars

Figure 2: Age standardised Hodgkin's Lymphoma incidence in the study population (cohort of UK population) by region. PYAR, person years at risk

Figure 3: Age standardised Hodgkin's Lymphoma incidence in the study population (cohort of UK population) by deprivation: in males and females (left panel) and in individuals aged ≤50 compared to >50 (right panel). PYAR, person years at risk

For peer review only

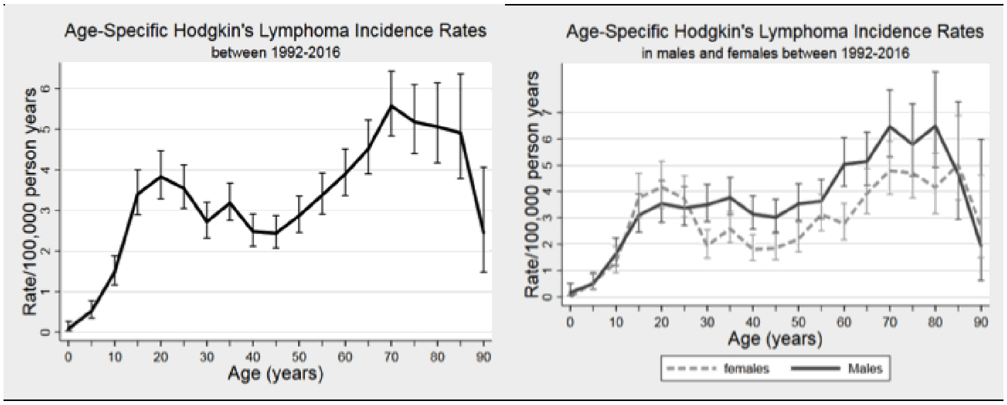

Figure 1: Age-specific Hodgkin's Lymphoma incidence in the study population (cohort of UK population): overall (left panel) and by sex (right panel), with 95% confidence interval bars

249x98mm (72 x 72 DPI)

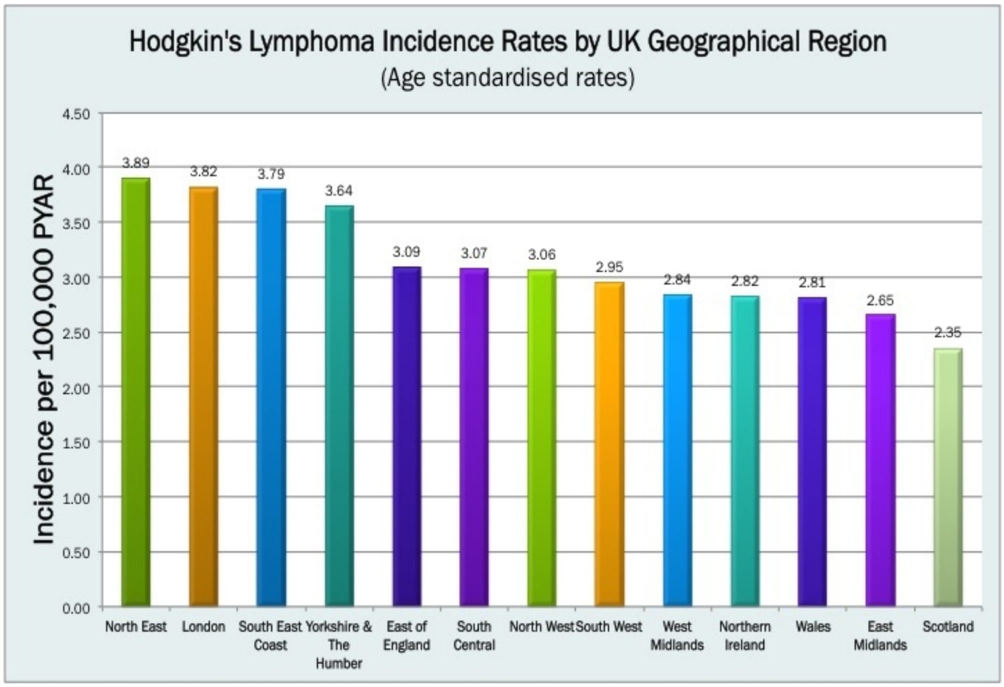

Figure 2: Age standardised Hodgkin's Lymphoma incidence in the study population (cohort of UK population) by region. PYAR, person years at risk

254x172mm (72 x 72 DPI)

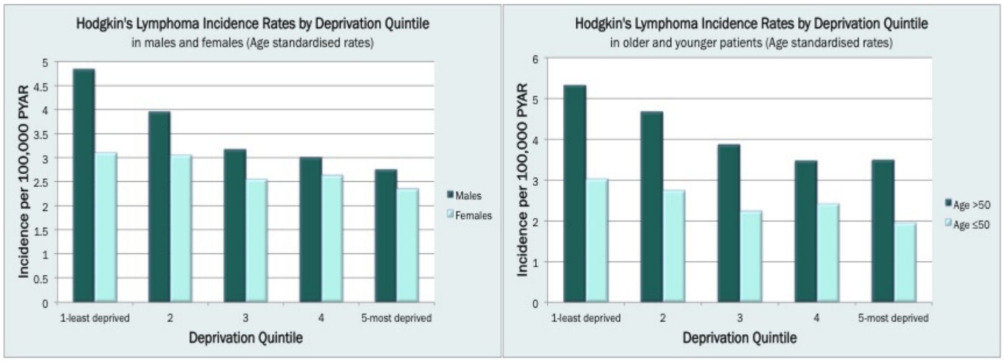

Figure 3: Age standardised Hodgkin’s Lymphoma incidence in the study population (cohort of UK population) by deprivation: in males and females (left panel) and in individuals aged ≤50 compared to >50 (right panel). PYAR, person years at risk

302x107mm (72 x 72 DPI)

Supplementary Tables:

Supplementary Table 1: Read codes for Hodgkin's Lymphoma

B61..00	Hodgkin's disease
B61..11	Hodgkin lymphoma
B610.00	Hodgkin's paragranuloma
B610100	Hodgkin's paragranuloma of lymph nodes of head, face, neck
B610300	Hodgkin's paragranuloma of intra-abdominal lymph nodes
B611.00	Hodgkin's granuloma
B611100	Hodgkin's granuloma of lymph nodes of head, face and neck
B612.00	Hodgkin's sarcoma
B612400	Hodgkin's sarcoma of lymph nodes of axilla and upper limb
B613.00	Hodgkin's disease, lymphocytic-histiocytic predominance
B613000	Hodgkin's, lymphocytic-histiocytic predominance unspec site
B613100	Hodgkin's, lymphocytic-histiocytic pred of head, face, neck
B613200	Hodgkin's, lymphocytic-histiocytic pred intrathoracic nodes
B613300	Hodgkin's, lymphocytic-histiocytic pred intra-abdominal node
B613500	Hodgkin's, lymphocytic-histiocytic pred inguinal and leg
B613600	Hodgkin's, lymphocytic-histiocytic pred intrapelvic nodes
B613700	Hodgkin's, lymphocytic-histiocytic predominance of spleen
B613800	Hodgkin's, lymphocytic-histiocytic pred of multiple sites
B613z00	Hodgkin's, lymphocytic-histiocytic predominance NOS
B614.00	Hodgkin's disease, nodular sclerosis
B614000	Hodgkin's disease, nodular sclerosis of unspecified site
B614100	Hodgkin's nodular sclerosis of head, face and neck
B614200	Hodgkin's nodular sclerosis of intrathoracic lymph nodes
B614300	Hodgkin's nodular sclerosis of intra-abdominal lymph nodes
B614400	Hodgkin's nodular sclerosis of lymph nodes of axilla and arm
B614700	Hodgkin's disease, nodular sclerosis of spleen

For peer review only

B614800	Hodgkin's nodular sclerosis of lymph nodes of multiple sites
B614z00	Hodgkin's disease, nodular sclerosis NOS
B615.00	Hodgkin's disease, mixed cellularity
B615000	Hodgkin's disease, mixed cellularity of unspecified site
B615100	Hodgkin's mixed cellularity of lymph nodes head, face, neck
B615200	Hodgkin's mixed cellularity of intrathoracic lymph nodes
B615500	Hodgkin's mixed cellularity of lymph nodes inguinal and leg
B615z00	Hodgkin's disease, mixed cellularity NOS
B616.00	Hodgkin's disease, lymphocytic depletion
B616000	Hodgkin's lymphocytic depletion of unspecified site
B616400	Hodgkin's lymphocytic depletion lymph nodes axilla and arm
B616700	Hodgkin's disease, lymphocytic depletion of spleen
B616800	Hodgkin's lymphocytic depletion lymph nodes multiple sites
B616z00	Hodgkin's disease, lymphocytic depletion NOS
B617.00	Nodular lymphocyte predominant Hodgkin lymphoma
B618.00	Nodular sclerosis classical Hodgkin lymphoma
B619.00	Mixed cellularity classical Hodgkin lymphoma
B61B.00	Lymphocyte-rich classical Hodgkin lymphoma
B61C.00	Other classical Hodgkin lymphoma
B61z.00	Hodgkin's disease NOS
B61z.11	Hodgkin lymphoma NOS
B61z000	Hodgkin's disease NOS, unspecified site
B61z100	Hodgkin's disease NOS of lymph nodes of head, face and neck
B61z200	Hodgkin's disease NOS of intrathoracic lymph nodes
B61z300	Hodgkin's disease NOS of intra-abdominal lymph nodes
B61z400	Hodgkin's disease NOS of lymph nodes of axilla and arm
B61z500	Hodgkin's disease NOS of lymph nodes inguinal region and leg
B61z700	Hodgkin's disease NOS of spleen
B61z800	Hodgkin's disease NOS of lymph nodes of multiple sites

For peer review only

B61zz00	Hodgkin's disease NOS
BBj..00	[M]Hodgkin's disease
BBj0.00	[M]Hodgkin's disease NOS
BBj1.00	[M]Hodgkin's disease, lymphocytic predominance
BBj1000	[M]Hodgkin,s disease, lymphocytic predominance, diffuse
BBj1100	[M]Hodgkin,s disease, lymphocytic predominance, nodular
BBj2.00	[M]Hodgkin's disease, mixed cellularity
BBj4.00	[M]Hodgkin's disease,lymphocytic depletion,diffuse fibrosis
BBj6.00	[M]Hodgkin's disease, nodular sclerosis NOS
BBj6000	[M]Hodgkin,s disease, nodular sclerosis, lymphocytic predom
BBj6100	[M]Hodgkin,s disease, nodular sclerosis, mixed cellularity
BBj6200	[M]Hodgkin,s disease, nodular sclerosis, lymphocytic deplet
BBj7.00	[M]Hodgkin's disease, nodular sclerosis, cellular phase
BBj9.00	[M]Hodgkin's granuloma
BBjz.00	[M]Hodgkin's disease NOS
ByuD000	[X]Other Hodgkin's disease
ZV10711	[V]Personal history of Hodgkin's disease

Supplementary Table 2: ICD10 codes for Hodgkin’s Lymphoma

Code	Term
C81	Hodgkin lymphoma
C81.0	Nodular lymphocyte predominant Hodgkin lymphoma
C81.1	Nodular sclerosis (classical) Hodgkin lymphoma
C81.2	Mixed cellularity (classical) Hodgkin lymphoma
C81.3	Lymphocyte depleted (classical) Hodgkin lymphoma
C81.4	Lymphocyte-rich (classical) Hodgkin lymphoma
C81.7	Other (classical) Hodgkin lymphoma

For peer review only

C81.9	Hodgkin lymphoma unspecified

For peer review only

Supplementary Table 3: Age-specific incidence rates of Hodgkin's Lymphoma by sex. CI, confidence interval; ASR, age standardised rate

Age group (years)	Incidence Rate Overall	95% CI	Incidence Rate Males	95% CI	Incidence Rate Females	95% CI	Incidence Rate Ratio (males / females)
0-4	0.09	0.03–0.27	0.17	0.05–0.52	0.00	–	
5-9	0.52	0.35–0.77	0.50	0.29–0.89	0.54	0.31–0.95	0.94
10-14	1.48	1.17–1.88	1.63	1.19–2.23	1.32	0.92–1.90	1.23
15-19	3.40	2.89–4.00	3.09	2.45–3.90	3.75	3.00–4.70	0.82
20-24	3.83	3.29–4.47	3.53	2.83–4.41	4.16	3.36–5.16	0.85
25-29	3.55	3.05–4.12	3.37	2.71–4.19	3.73	3.02–4.59	0.90
30-34	2.72	2.31–3.20	3.49	2.85–4.27	1.94	1.48–2.55	1.80
35-39	3.18	2.76–3.67	3.76	3.13–4.53	2.59	2.06–3.24	1.46
40-44	2.48	2.11–2.91	3.14	2.57–3.83	1.80	1.38–2.35	1.74
45-49	2.44	2.07–2.87	3.01	2.45–3.70	1.85	1.41–2.41	1.63
50-54	2.87	2.46–3.35	3.53	2.89–4.29	2.20	1.70–2.83	1.61
55-59	3.38	2.91–3.92	3.63	2.96–4.44	3.12	2.50–3.89	1.16
60-64	3.90	3.37–4.51	5.04	4.20–6.05	2.77	2.17–3.54	1.82
65-69	4.51	3.90–5.22	5.15	4.23–6.26	3.92	3.15–4.87	1.31
70-74	5.58	4.83–6.43	6.47	5.33–7.86	4.80	3.89–5.92	1.35
75-79	5.18	4.40–6.10	5.80	4.58–7.32	4.71	3.75–5.91	1.23
80-84	5.06	4.17–6.15	6.49	4.93–8.54	4.15	3.15–5.46	1.56
85-89	4.91	3.79–6.37	4.67	2.94–7.41	5.03	3.68–6.89	0.93
90+	2.45	1.48–4.07	1.93	0.62–5.99	2.63	1.49–4.63	0.73
**ASR**	**3.10**	**2.98–3.22**	**3.51**	**3.32–3.70**	**2.72**	**2.56–2.88**	

Supplementary Table 4: Crude and age-standardised Hodgkin's Lymphoma incidence rates in the UK by sex, age group, deprivation and geographical region. PYAR, person years at risk; ASR, age standardised rate; CI, confidence interval; IMD, index of multiple deprivation; yrs, age group in years

	Cases	PYAR	Incidence Rate per 100,000 PYAR	ASR per 100,000 PYAR	95%CI
Male	1331	39,039,332	3.41	3.51	3.32–3.70
Female	1071	39,529,340	2.71	2.72	2.56–2.88
IMD 1 (least deprived)	572	14,880,179	3.84	3.92	3.60–4.24
IMD 2	500	14,627,384	3.42	3.49	3.18–3.79
IMD 3	456	15,954,225	2.86	2.86	2.60–3.13
IMD 4	472	16,850,996	2.80	2.82	2.57–3.07
IMD 5 (most deprived)	402	16,256,652	2.47	2.55	2.30–2.80
North East England	53	1,419,931	3.73	3.89	2.84–4.94
Yorkshire/Humber	105	2,938,253	3.57	3.64	2.94–4.33
London	299	8,287,047	3.61	3.82	3.38–4.25
South East Coast	284	7,564,683	3.75	3.79	3.35–4.23
East of England	213	7,012,254	3.04	3.09	2.68–3.51
North West England	275	9,159,626	3.00	3.06	2.70–3.42
South West England	197	6,598,934	2.99	2.95	2.54–3.36
West Midlands	198	7,054,011	2.81	2.84	2.44–3.24
Wales	202	7,155,514	2.82	2.81	2.42–3.20
South Central England	256	8,482,798	3.02	3.07	2.70–3.45
East Midlands	80	3,042,062	2.63	2.65	2.07–3.23
Northern Ireland	70	2,597,902	2.69	2.82	2.16–3.48
Scotland	170	7,256,423	2.34	2.35	2.00–2.70
0-4yrs	3	3,507,576	0.09		
5-9yrs	24	4,620,605	0.52		
10-14yrs	68	4,590,771	1.48		
15-19yrs	147	4,321,501	3.40		
20-24yrs	162	4,226,413	3.83		
25-29yrs	170	4,794,244	3.55		
30-34yrs	147	5,402,587	2.72		
35-39yrs	187	5,877,364	3.18		
40-44yrs	151	6,089,433	2.48		
45-49yrs	145	5,943,893	2.44		
50-54yrs	159	5,541,281	2.87		
55-59yrs	172	5,094,300	3.38		
60-64yrs	179	4,592,514	3.90		
65-69yrs	181	4,009,358	4.51		
70-74yrs	189	3,390,115	5.58		
75-79yrs	144	2,779,666	5.18		
80-84yrs	102	2,015,256	5.06		
85-89yrs	57	1,160,678	4.91		
90+yrs	15	611,886	2.45		
**Overall ASR**				**3.10**	**2.98–3.22**

For peer review only

Reporting checklist for cohort study.

Based on the STROBE cohort guidelines.

Instructions to authors

Complete this checklist by entering the page numbers from your manuscript where readers will find each of the items listed below.

Your article may not currently address all the items on the checklist. Please modify your text to include the missing information. If you are certain that an item does not apply, please write "n/a" and provide a short explanation.

Upload your completed checklist as an extra file when you submit to a journal.

In your methods section, say that you used the STROBE cohort reporting guidelines, and cite them as:

von Elm E, Altman DG, Egger M, Pocock SJ, Gotzsche PC, Vandenbroucke JP. The Strengthening the Reporting of Observational Studies in Epidemiology (STROBE) Statement: guidelines for reporting observational studies.

For peer review only

		Reporting Item	Page Number
Title	#1a	Indicate the study’s design with a commonly used term in the title or the abstract	1
Abstract	#1b	Provide in the abstract an informative and balanced summary of what was done and what was found	3
Background / rationale	#2	Explain the scientific background and rationale for the investigation being reported	6
Objectives	#3	State specific objectives, including any prespecified hypotheses	6
Study design	#4	Present key elements of study design early in the paper	7, 8
Setting	#5	Describe the setting, locations, and relevant dates, including periods of recruitment, exposure, follow-up, and data collection	7, 8
Eligibility criteria	#6a	Give the eligibility criteria, and the sources and methods of selection of participants. Describe methods of follow-up.	7, 8
	#6b	For matched studies, give matching criteria and number of exposed and	n/a

		unexposed	
Variables	#7	Clearly define all outcomes, exposures, predictors, potential confounders, and effect modifiers. Give diagnostic criteria, if applicable	8, 9
Data sources / measurement	#8	For each variable of interest give sources of data and details of methods of assessment (measurement). Describe comparability of assessment methods if there is more than one group. Give information separately for for exposed and unexposed groups if applicable.	7, 8
Bias	#9	Describe any efforts to address potential sources of bias	7, 8, 9
Study size	#10	Explain how the study size was arrived at	7, 8
Quantitative variables	#11	Explain how quantitative variables were handled in the analyses. If applicable, describe which groupings were chosen, and why	8
Statistical methods	#12a	Describe all statistical methods, including those used to control for confounding	8, 9
	#12b	Describe any methods used to examine subgroups and interactions	9
	#12c	Explain how missing data were addressed	n/a
	#12d	If applicable, explain how loss to follow-up was addressed	7, 8
	#12e	Describe any sensitivity analyses	n/a
Participants	#13a	Report numbers of individuals at each stage of study—eg numbers potentially eligible, examined for eligibility, confirmed eligible, included in the study, completing follow-up, and analysed. Give information separately for for exposed and unexposed groups if applicable.	10
	#13b	Give reasons for non-participation at each stage	n/a
	#13c	Consider use of a flow diagram	n/a
Descriptive data	#14a	Give characteristics of study participants (eg demographic, clinical, social) and information on exposures and potential confounders. Give information separately for exposed and unexposed groups if applicable.	10
	#14b	Indicate number of participants with missing data for each variable of interest	n/a
	#14c	Summarise follow-up time (eg, average and total amount)	10

For peer review only

Outcome data	#15	Report numbers of outcome events or summary measures over time. Give information separately for exposed and unexposed groups if applicable.	10
Main results	#16a	Give unadjusted estimates and, if applicable, confounder-adjusted estimates and their precision (eg, 95% confidence interval). Make clear which confounders were adjusted for and why they were included	10, 11
	#16b	Report category boundaries when continuous variables were categorized	10, 11
	#16c	If relevant, consider translating estimates of relative risk into absolute risk for a meaningful time period	n/a
Other analyses	#17	Report other analyses done—e.g., analyses of subgroups and interactions, and sensitivity analyses	11
Key results	#18	Summarise key results with reference to study objectives	12
Limitations	#19	Discuss limitations of the study, taking into account sources of potential bias or imprecision. Discuss both direction and magnitude of any potential bias.	14
Interpretation	#20	Give a cautious overall interpretation considering objectives, limitations, multiplicity of analyses, results from similar studies, and other relevant evidence.	15, 16
Generalisability	#21	Discuss the generalisability (external validity) of the study results	13, 14
Funding	#22	Give the source of funding and the role of the funders for the present study and, if applicable, for the original study on which the present article is based	1, 2

The STROBE checklist is distributed under the terms of the Creative Commons Attribution License CC-BY. This checklist was completed on 17. January 2019 using https://www.goodreports.org/, a tool made by the EQUATOR Network in collaboration with Penelope.ai

For peer review only

BMJ Open

Socioeconomic deprivation and regional variation in Hodgkin's lymphoma incidence in the UK: A population-based cohort study of 10 million individuals

Journal:	*BMJ Open*
Manuscript ID	bmjopen-2019-029228.R1
Article Type:	Research
Date Submitted by the Author:	18-Apr-2019
Complete List of Authors:	Rafiq, Meena; University College London, Institute of Health Informatics<br>Hayward, Andrew; University College London, Institute of Epidemiology and Health Care<br>Warren-Gash, Charlotte; London School of Hygiene and Tropical Medicine, Non-communicable disease epidemiology<br>Denaxas, S; University College London, Institute of Health Informatics<br>Gonzalez-Izquierdo, Arturo; University College London, Institute of Health Informatics<br>Lyratzopoulos, Georgios ; University College London, Department of Epidemiology & Public Health, Health Behaviour Research Centre<br>Thomas, Sara; London School of Hygiene & Tropical Medicine, Faculty of Epidemiology & Population Health
**Primary Subject Heading**:	Oncology
Secondary Subject Heading:	Epidemiology, Haematology (incl blood transfusion)
Keywords:	Epidemiology < ONCOLOGY, Lymphoma < ONCOLOGY, Lymphoma < HAEMATOLOGY, Hodgkin's lymphoma, UK, deprivation

SCHOLARONE™
Manuscripts

Socioeconomic deprivation and regional variation in Hodgkin's lymphoma incidence in the UK: A population-based cohort study of 10 million individuals

Meena Rafiq[1], Andrew Hayward[2], Charlotte Warren-Gash[3], Spiros Denaxas[1], Arturo Gonzalez-Izquierdo[1], Georgios Lyratzopoulos[2] and Sara Thomas[3]

[1]Institute of Health Informatics, UCL, London, UK

[2]UCL institute of Epidemiology and Health Care, UCL, London, UK

[3]London School of Hygiene and Tropical Medicine, London, UK

Key Words: Hodgkin's lymphoma; epidemiology; UK; deprivation; regional variation

Corresponding Author: Dr Meena Rafiq**,** Institute of Health Informatics, University College London, 222 Euston Road, London, NW1 2DA, UK

Email: mrafiq@doctors.org.uk

Tel: +44 20 3549 5321

Word count:

Abstract:	296
Main Text:	3251
Number of Figures:	3
Number of Tables:	1

For peer review only - http://bmjopen.bmj.com/site/about/guidelines.xhtml

Abstract:

Objectives:

Hodgkin's Lymphoma (HL) is the commonest cancer in teenagers and young adults. This nationwide study conducted over a 25-year period in the UK investigates variation in HL incidence by age, sex, region and deprivation to identify trends and high-risk populations for HL development.

Design:

Population-based cohort study

Setting:

Clinical Practice Research Datalink (CPRD) electronic primary care records linked to Hospital Episode Statistics and Index of Multiple Deprivation data were used.

Participants:

Data on 10 million UK individuals from 1992–2016 were analysed.

Primary and secondary outcome measures:

Poisson models were used to explore differences in HL incidence by age, sex, region and deprivation. Age-specific HL incidence rates by sex and directly age-standardised incidence rates by region and deprivation group were calculated.

Results:

2,402 new cases of HL were identified over 78,569,436 person years. There was significant variation in HL incidence by deprivation group. Individuals living in the most affluent areas had HL incidence 60% higher than those living in the most deprived (incidence rate ratios (IRR) 1.60, 95% confidence interval 1.40–1.83), with strong evidence of a marked linear trend towards increasing HL incidence with decreasing deprivation (p=<0.001). There was significant regional variation in HL incidence across the UK, which persisted after adjusting for age, sex and deprivation (IRR 0.80–1.42, p=<0.001).

For peer review only

Conclusions:

This study identified high-risk regions for HL development in the UK and observed a trend towards higher incidence of HL in individuals living in less deprived areas. Consistent with findings from other immune-mediated diseases, this study supports the hypothesis that an affluent childhood environment may predispose to development of immune-related neoplasms, potentially through fewer immune challenges interfering with immune maturation in early life. Understanding the mechanisms behind this immune dysfunction could inform prevention, detection and treatment of HL and other immune diseases.

Article Summary:

Strengths and limitations of the study

- Our population-based data covered a large representative sample of over 10 million individuals in the UK over a 25-year period with 78 million years of follow up.
- We used UK primary care electronic health records linked to secondary care data and Index of Multiple Deprivation data to improve capture of Hodgkin's lymphoma diagnoses and allow analysis of geographical and deprivation based trends.
- Data from the Clinical Practice Research Datalink (CPRD) used in this study have been demonstrated to be generalisable to the UK population across a number of demographics.
- Data in this study were not linked to the National Cancer Register (NCR), which is a potential limitation; however lymphoma diagnosis in CPRD has been validated in previous studies and shown to have high concordance with the NCR.

For peer review only - http://bmjopen.bmj.com/site/about/guidelines.xhtml

- This is a cohort study of a representative sample of the UK population and not the whole UK population.

Financial support:

Dr Meena Rafiq and the work presented in this paper are funded by a National Institute for Health Research (NIHR) in-practice clinical fellowship (IPF-2017-11-011). This article presents independent research funded by the NIHR. The views expressed are those of the author(s) and not necessarily those of the NHS, the NIHR or the Department of Health. GL is supported by Cancer Research UK Advanced Clinician Scientist Fellowship (C18081/A18180).

Conflict of interest: The authors declare no potential conflicts of interest.

Contributors: MR, AH, CWG and ST contributed to the conception and design of the study, planning of analyses, interpretation of results and writing the manuscript. MR, SD and AGI contributed to the planning of the analyses, extracting the data and performing the statistical analyses. GL contributed to study design and interpretation of results. All authors have read and approved the final manuscript.

Data sharing statement CPRD data on which the study was based is held securely by University College London under the CPRD data access licence (https://www.cprd.com/dataAccess/).

Acknowledgements:

We thank the patient and public advisers who assisted in the design and conception of this study.

For peer review only - http://bmjopen.bmj.com/site/about/guidelines.xhtml

Introduction:

Hodgkin's Lymphoma (HL) is the commonest cancer in teenagers and young adults worldwide (1, 2). In the UK 2,100 new cases of HL are diagnosed each year, but little is known about the distribution of these cases in the UK population or if there are any high-risk groups. International studies have identified that HL incidence varies considerably between countries, with higher rates observed in high-income countries (3-7). This pattern is also seen within countries, with US studies showing higher rates in more affluent regions and geographical variation in HL incidence between different states (8). Few UK studies have investigated HL incidence patterns by socioeconomic deprivation (9-12) and region (12-14) and the results have been conflicting and inconclusive. Additionally, to our knowledge there have been no recent studies investigating patterns of HL incidence in the UK population since 2010. Understanding how HL incidence varies between different geographical regions in the UK and identifying high-risk populations may provide clues to the underlying aetiology of the disease and inform future research directions. We aimed to conduct a population-based cohort study of 10 million individuals over a 25-year period using linked primary and secondary care electronic health records to investigate variation in HL incidence within the UK by age, sex, geographic region and deprivation.

For peer review only

Materials and methods:

Data sources and study population

Data were obtained from the UK Clinical Practice Research Datalink (CPRD), linked to Hospital Episode Statistic (HES) inpatient data and Index of Multiple Deprivation

For peer review only - http://bmjopen.bmj.com/site/about/guidelines.xhtml

(IMD) data. CPRD is an electronic health record database containing prospectively collected pseudo-anonymised data from UK primary care consultations. It is the largest source of longitudinal primary care data, holding information on 22 million patients representing approximately 9% of the UK population (in 2013) (15). This database has been shown to be largely representative of the UK population across a number of demographics including age, sex and ethnicity (15). Data are available from 1987 onwards when CPRD was first established. Practices contributing to CPRD are regularly audited to ensure high data quality and that 95% of prescribing and morbidity events are captured before practices are declared 'up-to-standard' (UTS) for research purposes (16). HES data provide additional information from hospital attendances in England. IMD scores represent a composite ecological (small-area based) measure of the socioeconomic status of a patient, based on the income, employment, disability, educational attainment and other attributes of the LSOA (Local Super Output Area) of a patients' residence. The latter typically comprise populations between 1,000 and 3,000 residents. All patients had an aggregate IMD score pertaining to the LSOA of their own residence (0.1% of population) or that of their general practice (99.9%) taken from the earliest available linked IMD dataset (2004 for patient-level and 2009 for practice-level).

The study population comprised patients actively registered with a CPRD practice between January 1992 and December 2016 who did not have a pre-existing diagnosis of HL. In accordance with previous studies, eligible follow-up time in days for each patient was commenced from one year after the patient registered with the practice (to avoid capturing past diagnoses recorded retrospectively in the few months after new patient registration) (17), or from when CPRD classified the GP practice to be 'up-to-standard' (UTS) if this occurred later. Active follow-up ended when a patient received a diagnosis of HL, died, left a CPRD practice or at the last data collection date for participating practices, whichever occurred earlier.

For peer review only - http://bmjopen.bmj.com/site/about/guidelines.xhtml

For peer review only

Classification of outcome and exposure

Data were obtained on HL diagnoses coded using Read codes (in CPRD) or the International Classifications of Diseases, 10th Revision codes (ICD-10, in HES) (Supplementary Table S1 and S2); age and date of diagnosis; area of residence by Strategic Health Authority (SHA) region; deprivation using IMD quintiles; date of birth and sex.

Statistical analysis

For each new case of HL, the year and age at diagnosis were determined and the patient was counted as an incident case for that calendar year and age group. The duration of active follow-up in CPRD for each individual in the study population was then calculated and used to calculate the total person years at risk (PYAR) and to estimate crude HL incidence rates per 100,000 PYAR.

Age-specific HL incidence rates were calculated in 5-year age bands, first for persons and then stratified by sex. Age-standardised incidence rates were estimated by the direct method using the European standard population for each region and deprivation quintile. Poisson regression was used to model HL incidence rate ratios (IRRs) for region, deprivation, age and sex independently before adjusting for other variables. East of England was used as the reference category for region, as the region with age-standardised incidence estimate that was closest to the national average(18). Deprivation was initially included as a categorical variable in the regression analysis to calculate IRRs and then subsequently we assessed for a linear trend by deprivation quintile, first by estimating the linear effect of deprivation using likelihood ratio tests, and then investigating departure from linearity by

For peer review only

comparing models in which deprivation was added as a non-linear vs. a linear term. Additionally, incidence rates by deprivation were examined to see if any variation persisted after adjusting for trends in region, and vice versa to see if trends in region were observed after adjusting for deprivation as a categorical variable. Adjusted models were also adjusted for age and sex.

HL has a bimodal age-specific incidence pattern with the first peak occurring between 15-34 years and a second peak between 70-84 years (18). Previous studies have suggested HL in individuals aged <50 and >50 is likely to have different aetiological factors. We therefore performed pre-specified subgroup analyses by sex (male vs. female) and age (≤50 vs. >50 years). We additionally examined interactions between exposure variables in the final model, particularly given potential variation in risk of HL by age and sex (18) i.e. age group by sex, age group by deprivation, age group by region, sex by deprivation and sex by region. Deprivation group was treated as a categorical variable in interaction terms. Analyses were performed using Stata (version 15; StataCorp, College Station, TX, USA).

Patient and Public Involvement

The development of the research question for this study and aspects of the study design, particularly the subgroup analysis of the outcome by sex and age group, were informed by discussions with Hodgkin's lymphoma patients' and their friends and relatives. The research focus of this study reflects their experiences and expressed research priorities in this field. Results will be shared with patient and public advisers and publicised on the CPRD website with details of the open-access paper.

For peer review only - http://bmjopen.bmj.com/site/about/guidelines.xhtml

Results:

There were 2,402 new diagnoses of HL identified over the 25-year study period (78,569,436 person years of follow-up) with an overall HL incidence of 3.06 cases per 100,000 PYAR (95% confidence interval (CI) 2.94–3.18). 47.2% of cases were identified using CPRD alone, 16.9% were identified using HES alone and 35.9% were identified in both datasets. Age-specific HL incidence showed a bimodal distribution with an initial peak at ages 20–24 years followed by a second peak at ages 70–74 years characteristic of HL incidence in high-income countries (Figure 1, Supplementary Table S3 and S4). Incidence was higher in older adults compared to those aged ≤50 (4.12 cases per 100,000 PYAR, 95%CI 3.89–4.36 vs. 2.46 cases per 100,000 PYAR, 95%CI 2.32–2.59) and was higher in males than in females in all age groups (with an overall IRR for males vs. females of 1.26, 95%CI 1.16-1.36 and age-specific IRRs ranging from 1.16–1.82) except for 15–29 years when incidence in females exceeded that of males (age-specific IRRs 0.82-0.90) and at the extremes of the age range where the number of cases were small (Figure 1, Supplementary Table S3). Of the 2,402 incident cases of HL, 52.8% were identified in HES (407 not in CPRD), 83.1% in CPRD (1,133 not in HES) and 35.9% were identified in both.

Regional variation

Age-standardised incidence rates showed variation in HL incidence across the UK with the North East of England having the highest rates (3.89 cases per 100,000 PYAR) and Scotland having the lowest (2.35 cases per 100,000 PYAR) (Figure 2, Supplementary Table S4). Multivariable Poisson regression revealed strong evidence for an association between geographical region and HL incidence (Table 1), which persisted after adjusting for deprivation, age and sex (p=<0.001). Subgroup analysis showed that regional variation in HL incidence was observed in both males and females, but was limited to individuals aged over 50 years, without evidence for

For peer review only - http://bmjopen.bmj.com/site/about/guidelines.xhtml

an association between region and HL incidence demonstrated in the younger age group (p=0.23).

Socioeconomic deprivation

There was strong evidence for an association between HL incidence and deprivation (p=<0.001), with age-standardised incidence being highest in the most affluent population groups and lowest in the most deprived (3.92 cases per 100,000 PYAR vs. 2.55 cases per 100,000 PYAR, Supplementary Table S4). Poisson regression showed that the least deprived group had HL incidence rates over 50% higher than the most deprived one, after adjusting for other factors (IRR = 1.55 and 1.63 for individuals aged <50 vs. ≥50 respectively, Table 1). The strong evidence of a marked linear trend towards lower rates of HL incidence with increasing deprivation persisted after adjusting for region and was observed across both sexes and when analysing young and old adults separately (Figure 3, Table 1).

Interaction analysis

Further exploring the subgroup analysis outlined above, there was no evidence that regional differences in HL incidence varied by age or sex ($P_{interaction}$= 0.40 and 1.00 respectively). Additionally, there was no evidence that the association between deprivation and HL risk varied by age or sex ($P_{interaction}$= 0.57 and 0.39 respectively). The characteristic bimodal age-specific HL incidence pattern was observed in both males and females, and the association between age and HL incidence did not vary by sex ($P_{interaction}$= 0.16).

For peer review only - http://bmjopen.bmj.com/site/about/guidelines.xhtml

Discussion:

This is the largest study to date investigating variability in HL incidence by age, sex, deprivation and sub-national geography. It uses linked electronic primary care records over a 25-year period in a representative cohort of the UK population. We found strong evidence that lower level of deprivation is associated with a higher incidence of HL, an association observed across age groups. There was considerable variation in HL incidence by UK geographical region, and these differences persisted after sex, age and deprivation were taken into account.

Comparison with the literature:

The bimodal age-specific HL incidence pattern described in this study is consistent with findings from other high-income countries, including previous studies in the UK (3, 5-7, 18-22). Higher incidence of HL in males except between ages 15-29 has also been observed in previous UK studies, which found higher incidence in females aged 15-24 (18, 21). When looking at the association with deprivation previous studies have shown heterogeneous outcomes. A previous UK study found that HL incidence in males between 2006-2010 was greater in more deprived areas, without finding associations between deprivation and HL in the earlier study era (1996-2006) in either sex (9). Another study investigating the distribution of childhood cancers in the UK between 1969-1993 found HL incidence in children aged 0-9 was greater in more deprived areas (11). In contrast, two previous studies conducted in parts of England and Wales reported higher HL incidence with higher socioeconomic status in individuals aged 0-24 (10, 12), concordant with our study findings, which are based on a population of 10 million people followed-up over a longer time period, broader geographic area and including patients other than young adults, adolescents and children. The observation of inverse socioeconomic gradients for incidence of HL in older adults in our study is a finding which to our knowledge has not been previously

For peer review only

reported. With regards to regional variation the previous literature has also been conflicting. Quinn et al. and McKinney et al. reported no clear geographical variation in HL incidence rates within the UK population (13, 14), though Alston et al. reported strong evidence that HL incidence varied by UK region, with greater incidence in London and the South East of England among individuals aged 0-24 years (12). These findings concord with those of our study, which demonstrated significant regional variation in HL incidence across the UK with greater incidence in London and the South East. Regional variations were present in both males and females, but were limited to older adults, although this may reflect power limitations rather than a true lack of an effect in younger adults.

Strengths and limitations

The main strengths of our study are that it is a large population-based study of more than 10 million individuals and has a long follow-up. HL is a relatively rare disease and the sample size and follow-up length allow for smaller effect sizes and interactions that could be missed in smaller studies to be detected. This is particularly important due to the growing evidence for two potentially separate aetiological pathways underlying HL incidence in young and older adults, and therefore the need for them to be analysed independently (3, 4, 23). A further advantage of this study was the use of CPRD data with regional information, linked to HES and deprivation data. CPRD has wide national coverage and has been demonstrated to be representative of the UK population across a number of demographics making the results generalisable to the UK population (15).

The main limitation of this study is the that it did not have access to linked data from the UK National Cancer Registry (NCR), which can be considered to represent the gold standard for estimating HL incidence. This could result in potential misclassification of cases and controls in this study and subsequent underestimation

For peer review only

For peer review only - http://bmjopen.bmj.com/site/about/guidelines.xhtml

of effect estimates. Previous concordance studies have demonstrated that HL diagnoses have high validity in CPRD when compared to the NCR (positive predictive value for lymphoma 89.6%, sensitivity 97.3%) and any such effect is therefore unlikely to have materially affected the findings (24). Additionally, CPRD has established use in cancer epidemiology in the literature (24-27) and previous population-based cohort studies have demonstrated the feasibility of Hodgkin's lymphoma research using CPRD (28-30). Outcome misclassification in this study was further reduced through use of HES-linked data, which improved validity of HL diagnoses by supplementing GP records with hospital data to capture cases that might have been missed in CPRD. Data was also not available on HL subtype and EBV positivity status, which would be informative for subgroup analysis to assess if trends in deprivation varied by histological group. This could be explored in future studies. Another limitation is the use of routinely collected data with potential misclassification of an individual's deprivation group. Deprivation was determined using IMD which is based on the postcode of the patients residence or registered GP practice and not on individual-level characteristics. As there may be variation in deprivation within a postcode, especially in highly diverse inner-city areas, this could result in non-differential misclassification of deprivation and underestimation of any effects. Additionally, the deprivation quintile and region captured from the dataset and used in the study may not represent childhood deprivation groups and region of residence, which may be more appropriate if early-life exposures are involved in the etiology of HL. The earliest available linked IMD scores (2004 for patient level and 2009 for practice-level) were used in this study to estimate deprivation. This assumes both that an individuals IMD status remains stable throughout their life, and that the IMD quintile of a postcode remains stable over time. Both of these assumptions may not be true as individuals can move between deprivation quintiles and areas may undergo gentrification over time. Population movement also means an individuals

For peer review only

For peer review only - http://bmjopen.bmj.com/site/about/guidelines.xhtml

childhood residence may differ from their current regional residence, which could dilute any regional variation observed in HL incidence.

Implications

The bimodal incidence pattern and differences in regional variation between younger versus older adults supports the hypothesis that there may be different aetiological pathways involved in the development of HL in these age groups (3, 4, 19). This is further supported by evidence from previous studies for different distributions of the histological subtypes of HL between the two age groups (5, 7, 8, 23, 31-33). Consideration should be given to investigating HL aetiology separately in these age groups in future studies to identify potential different contributory factors that could be masked when analysing the population as a whole. Additionally, the existence of potentially different pathophysiology could have important implications for targeting and response to treatment regimens and in disease monitoring and detection. The peak in disease incidence in young adult females is characteristic of a number of immune-related conditions, including multiple sclerosis, rheumatoid arthritis and lupus (34-36). Similarities between incidence patterns for these diseases could suggest a common predisposing factor in early life that interferes with immune regulation and promotes development of immune-related diseases in young adults.

The trend towards increased HL incidence with increased affluence was replicated across three separate UK databases and is consistent with findings from US studies (8, 10, 12). Concordance between these findings add further support for this being a true association. This trend has been previously well established in ecological studies making comparisons between countries with very different levels of deprivation (3-7). Within country differences in deprivation tend to be much smaller than those seen between countries. Our results could suggest that even small

For peer review only

For peer review only - http://bmjopen.bmj.com/site/about/guidelines.xhtml

increases in community deprivation levels may elevate an individual's risk of developing HL. A proposed explanation for this association is that children in affluent households with less overcrowding and cleaner childhood environments consequently have delayed exposure to infectious agents and fewer immune challenges in early-life to stimulate immune development and regulation (37-42). This predisposes them to develop immune related conditions. This phenomenon has been demonstrated for other haematological malignancies, including leukaemia, where low infection burden and lack of microbial exposure in early life were found to result in immune system malfunction and were associated with increased risk of developing subsequent leukaemia(43). Observation of this trend in older adults is less likely to be explained by childhood exposures. HL aetiology could be multifactorial with childhood exposures predisposing individuals, but in the absence of other promoting factors in early-life, onset of HL is delayed until later adulthood. This should be further explored in future studies to identify contributory factors underlying the association in older adults.

Regional variation in HL incidence was observed after adjusting for deprivation differences in older adults. This indicates that other factors that vary geographically in these regions are contributing to increased HL incidence in this age group. Geographical clustering of HL cases has been previously reported in both the UK and USA (8, 12, 44-47), which could support the role for an environmental factor underlying increased rates in these regions. Other possible contributory factors include regional differences in ethnicity and clustering of predisposing or protective genotypes. Further studies are required to investigate the role of these different factors in regional variation in UK HL incidence.

Conclusion

For peer review only

This study of over 10 million individuals based on nationwide primary care data found strong evidence for regional variation in HL incidence across the UK that cannot be explained by geographical differences in deprivation. More affluent individuals within the UK population have a significantly higher risk of developing HL in both younger and older adults. This trend has been observed for other immune-mediated diseases. The findings are consistent with the hypothesis that an affluent childhood environment may predispose to development of immune-related conditions, possibly through fewer immune challenges interfering with the maturation of the immune system. Further understanding the responsible pathophysiological mechanisms could inform prevention, detection and treatment of HL and other immune conditions.

For peer review only

References:

1. Bleyer WA. Cancer in older adolescents and young adults: epidemiology, diagnosis, treatment, survival, and importance of clinical trials. Med Pediatr Oncol. 2002;38(1):1-10.
2. Alarcon PA. Pediatric Hodgkin Lymphoma http://emedicine.medscape.com/article/987101-overview - a5: Medscape, medicine; 2017 [
3. MACMAHON B. Epidemiological evidence of the nature of Hodgkin's disease. Cancer. 1957;10(5):1045-54.
4. MacMahon B. Epidemiology of Hodgkin's disease. Cancer Res. 1966;26(6):1189-201.
5. Correa P, O'Conor GT. Epidemiologic patterns of Hodgkin's disease. Int J Cancer. 1971;8(2):192-201.
6. Harris NL. The many faces of Hodgkin's disease around the world: what have we learned from its pathology? Ann Oncol. 1998;9 Suppl 5:S45-56.
7. Parkin D, Whelan S, Ferlay J, Teppo L, Thomas D. Cancer incidence in five continents, Vol. VIII, International Agency for Research on Cancer.(IARC Scientific Publication 155). Lyon, France: IARC Press; 2002.
8. Glaser SL. Regional variation in Hodgkin's disease incidence by histologic subtype in the US. Cancer. 1987;60(11):2841-7.
9. Network NCI. Cancer by Deprivation in England. Incidence, 1996-2010. Mortality, 1997-2011. 2014.
10. Alexander FE, Ricketts TJ, McKinney PA, Cartwright RA. Community lifestyle characteristics and incidence of Hodgkin's disease in young people. Int J Cancer. 1991;48(1):10-4.
11. Elliott A. Committee on Medical Aspects of Radiation in the Environment (COMARE). 11th report. The distribution of childhood leukaemia and other childhood cancers in Great Britain 1969–1993. . 2006.
12. Alston RD, Rowan S, Eden TO, Moran A, Birch JM. Cancer incidence patterns by region and socioeconomic deprivation in teenagers and young adults in England. Br J Cancer. 2007;96(11):1760-6.
13. Quinn M, Wood H, Cooper N, Rowan S. Cancer atlas of the United Kingdom and Ireland 1991–2000. Studies on medical and population subjects. 2005;68.
14. McKinney PA, Alexander FE, Ricketts TJ, Williams J, Cartwright RA. A specialist leukaemia/lymphoma registry in the UK. Part 1: Incidence and geographical distribution of Hodgkin's disease. Leukaemia Research Fund Data Collection Study Group. Br J Cancer. 1989;60(6):942-7.

For peer review only

For peer review only - http://bmjopen.bmj.com/site/about/guidelines.xhtml

15. Herrett E, Gallagher AM, Bhaskaran K, Forbes H, Mathur R, van Staa T, et al. Data Resource Profile: Clinical Practice Research Datalink (CPRD). Int J Epidemiol. 2015;44(3):827-36.

16. Digital N. Hospital Episode Statistics http://content.digital.nhs.uk/hes2017 [

17. Lewis JD, Bilker WB, Weinstein RB, Strom BL. The relationship between time since registration and measured incidence rates in the General Practice Research Database. Pharmacoepidemiol Drug Saf. 2005;14(7):443-51.

18. Cancer Research UK. Hodgkin lymphoma incidence statistics May 2018 [Available from: http://www.cancerresearchuk.org/health-professional/cancer-statistics/statistics-by-cancer-type/hodgkin-lymphoma - heading-Zero.

19. Hjalgrim H. On the aetiology of Hodgkin lymphoma. Dan Med J. 2012;59(7):B4485.

20. Clemmesen J. Statistical studies in the aetiology of malignant neoplasms. Acta Pathol Microbiol Scand Suppl. 1974;Suppl 247:1-266.

21. Roman E, Smith AG. Epidemiology of lymphomas. Histopathology. 2011;58(1):4-14.

22. Smith A, Crouch S, Lax S, Li J, Painter D, Howell D, et al. Lymphoma incidence, survival and prevalence 2004–2014: sub-type analyses from the UK's Haematological Malignancy Research Network. British journal of cancer. 2015;112(9):1575.

23. Newell GR, Cole SR, Miettinen OS, MacMahon B. Age differences in the histology of Hodgkin's disease. Journal of the National Cancer Institute. 1970;45(2):311-7.

24. Boggon R, van Staa TP, Chapman M, Gallagher AM, Hammad TA, Richards MA. Cancer recording and mortality in the General Practice Research Database and linked cancer registries. Pharmacoepidemiol Drug Saf. 2013;22(2):168-75.

25. Dregan A, Moller H, Murray-Thomas T, Gulliford MC. Validity of cancer diagnosis in a primary care database compared with linked cancer registrations in England. Population-based cohort study. Cancer Epidemiol. 2012;36(5):425-9.

26. Arhi CS, Bottle A, Burns EM, Clarke JM, Aylin P, Ziprin P, et al. Comparison of cancer diagnosis recording between the Clinical Practice Research Datalink, Cancer Registry and Hospital Episodes Statistics. Cancer Epidemiol. 2018;57:148-57.

27. Margulis AV, Fortuny J, Kaye JA, Calingaert B, Reynolds M, Plana E, et al. Validation of Cancer Cases Using Primary Care, Cancer Registry, and Hospitalization Data in the United Kingdom. Epidemiology (Cambridge, Mass). 2018;29(2):308.

For peer review only

For peer review only - http://bmjopen.bmj.com/site/about/guidelines.xhtml

28. Strongman H, Brown A, Smeeth L, Bhaskaran K. Body mass index and Hodgkin's lymphoma: UK population-based cohort study of 5.8 million individuals. British journal of cancer. 2019:1.

29. Castellsague J, Kuiper JG, Pottegård A, Berglind IA, Dedman D, Gutierrez L, et al. A cohort study on the risk of lymphoma and skin cancer in users of topical tacrolimus, pimecrolimus, and corticosteroids (Joint European Longitudinal Lymphoma and Skin Cancer Evaluation–JOELLE study). Clinical epidemiology. 2018;10:299.

30. Gelfand JM, Berlin J, Van Voorhees A, Margolis DJ. Lymphoma rates are low but increased in patients with psoriasis: results from a population-based cohort study in the United Kingdom. Archives of dermatology. 2003;139(11):1425-9.

31. Franssila KO, Heiskala MK, Heiskala HJ. Epidemiology and histopathology of Hodgkin's disease in Finland. Cancer. 1977;39(3):1280-8.

32. Henderson BE, Dworsky R, Pike MC, Baptista J, Menck H, Preston-Martin S, et al. Risk factors for nodular sclerosis and other types of Hodgkin's disease. Cancer Res. 1979;39(11):4507-11.

33. Cozen W, Katz J, Mack TM. Risk patterns of Hodgkin's disease in Los Angeles vary by cell type. Cancer Epidemiol Biomarkers Prev. 1992;1(4):261-8.

34. Whitacre CC, Reingold SC, O'Looney PA. A gender gap in autoimmunity. Science. 1999;283(5406):1277-8.

35. Jacobson DL, Gange SJ, Rose NR, Graham NM. Epidemiology and estimated population burden of selected autoimmune diseases in the United States. Clin Immunol Immunopathol. 1997;84(3):223-43.

36. Beeson PB. Age and sex associations of 40 autoimmune diseases. Am J Med. 1994;96(5):457-62.

37. Glaser SL, Lin RJ, Stewart SL, Ambinder RF, Jarrett RF, Brousset P, et al. Epstein-Barr virus-associated Hodgkin's disease: epidemiologic characteristics in international data. Int J Cancer. 1997;70(4):375-82.

38. Chang ET, Zheng T, Weir EG, Borowitz M, Mann RB, Spiegelman D, et al. Childhood social environment and Hodgkin's lymphoma: new findings from a population-based case-control study. Cancer Epidemiol Biomarkers Prev. 2004;13(8):1361-70.

39. Glaser SL, Clarke CA, Nugent RA, Stearns CB, Dorfman RF. Social class and risk of Hodgkin's disease in young-adult women in 1988-94. Int J Cancer. 2002;98(1):110-7.

40. Gutensohn N, Cole P. Epidemiology of hodgkin's disease in the young. Int J Cancer. 1977;19(5):595-604.

For peer review only

For peer review only - http://bmjopen.bmj.com/site/about/guidelines.xhtml

41. Mueller N. Hodgkin's disease. In: Schottendfeld D, Fraumeni JF Jr, eds. Cancer epidemiology and prevention. New York: Oxford University Press; 1996.

42. Mueller N, Grufferman S. The epidemiology of Hodgkin's disease. In: Mauch PM, Armitage JO, Diehl V, et al, eds. Hodgkin's disease. Philadelphia: Lippincott Williams & Wilkins; 1999.

43. Greaves M. A causal mechanism for childhood acute lymphoblastic leukaemia. Nature Reviews Cancer. 2018:1.

44. Barnes N, Cartwright RA, O'Brien C, Roberts B, Richards ID, Bird CC. Spatial patterns in electoral wards with high lymphoma incidence in Yorkshire health region. Br J Cancer. 1987;56(2):169-72.

45. Cartwright RA, Watkins G. Epidemiology of Hodgkin's disease: a review. Hematol Oncol. 2004;22(1):11-26.

46. Smith PG. Current assessment of "case clustering" of lymphomas and leukemias. Cancer. 1978;42(2 Suppl):1026-4.

47. Alexander FE, Williams J, McKinney PA, Ricketts TJ, Cartwright RA. A specialist leukaemia/lymphoma registry in the UK. Part 2: Clustering of Hodgkin's disease. Br J Cancer. 1989;60(6):948-52.

For peer review only

For peer review only - http://bmjopen.bmj.com/site/about/guidelines.xhtml

Table 1: Hodgkin's lymphoma risk by sex, socioeconomic status and geographical region

	Adjusted IRR (95%CI)¥				
Risk factors	Study Population	≤50 years	>50 years	Males	Females
Sex					
Male	1.30 (1.20-1.41)	1.23 (1.10-1.38)	1.38 (1.23-1.55)		
p value	<0.001	<0.001	<0.001		
Region					
East of England	*ref*	*ref*	*ref*	*ref*	*ref*
North East England	1.42 (1.05-1.93)	0.82 (0.48-1.40)	2.05 (1.40-3.01)	1.49 (1.00-2.24)	1.34 (0.84-2.13)
Yorkshire/Humber	1.32 (1.04-1.68)	1.11 (0.78-1.58)	1.55 (1.12-2.13)	1.48 (1.08-2.01)	1.15 (0.79-1.66)
London	1.29 (1.08-1.54)	1.15 (0.90-1.48)	1.45 (1.13-1.87)	1.25 (0.99-1.59)	1.33 (1.02-1.73)
South East Coast	1.23 (1.03-1.48)	1.24 (0.96-1.59)	1.24 (0.97-1.59)	1.19 (0.94-1.51)	1.29 (0.99-1.69)
North West England	1.07 (0.89-1.28)	1.05 (0.82-1.36)	1.07 (0.83-1.39)	1.13 (0.89-1.43)	0.99 (0.75-1.31)
South West England	1.06 (0.87-1.29)	1.08 (0.82-1.43)	1.04 (0.78-1.37)	1.05 0.81-1.36)	1.07 (0.80-1.43)
West Midlands	1.00 (0.82-1.21)	0.90 (0.68-1.20)	1.10 (0.83-1.44)	0.96 (0.74-1.26)	1.04 (0.78-1.40)
Wales	0.97 (0.80-1.18)	1.08 (0.83-1.42)	0.87 (0.65-1.15)	0.96 (0.74-1.25)	0.98 (0.73-1.32)
South Central England	0.96 (0.80-1.15)	0.96 (0.75-1.24)	0.95 (0.73-1.23)	0.87 (0.68-1.11)	1.08 (0.82-1.42)
East Midlands	0.95 (0.73-1.23)	1.10 (0.78-1.54)	0.79 (0.53-1.17)	0.94 (0.67-1.34)	0.96 (0.65-1.42)
Northern Ireland	0.90 (0.68-1.17)	0.78 (0.53-1.15)	1.02 (0.70-1.48)	0.90 (0.63-1.29)	0.90 (0.60-1.06)
Scotland	0.80 (0.66-0.98)	0.89 (0.68-1.17)	0.71 (0.53-0.96)	0.82 (0.63-1.08)	0.78 (0.57-1.06)
p value	<0.001	0.23	<0.001	0.002	0.03
IMD quintile					
5 (most deprived)	*ref*	*ref*	*ref*	*ref*	*ref*
4	1.10 (0.96-1.26)	1.20 (1.00-1.45)	1.01 (0.83-1.23)	1.11 (0.92-1.34)	1.09 (0.89-1.33)
3	1.15 (1.00-1.32)	1.12 (0.92-1.36)	1.18 (0.97-1.44)	1.21 (1.00-1.47)	1.08 (0.88-1.33)
2	1.35 (1.18-1.55)	1.37 (1.13-1.66)	1.33 (1.10-1.62)	1.45 (1.21-1.75)	1.25 (1.02-1.52)
1 (least deprived)	1.60 (1.40-1.83)	1.55 (1.29-1.88)	1.63 (1.35-1.97)	1.87 (1.57-2.24)	1.31 (1.07-1.61)
p value	<0.001*	<0.001*	<0.001*	<0.001*	0.003*

Adjusted IRR, ¥incidence rate ratio adjusted for age, sex, region and IMD quintile; IMD, Index of Multiple Deprivation; CI, confidence interval; p, p value from likelihood-ratio test; * p value from test for linear trend; ref, reference group (East of England used as the reference category as the region with age-standardised incidence estimate that was closest to the national average)

For peer review only

Figure Legends:

Figure 1: Age-specific Hodgkin's Lymphoma incidence in the study population (cohort of UK population): overall (left panel) and by sex (right panel), with 95% confidence interval bars

Figure 2: Age standardised Hodgkin's Lymphoma incidence in the study population (cohort of UK population) by region. PYAR, person years at risk

Figure 3: Age standardised Hodgkin's Lymphoma incidence in the study population (cohort of UK population) by deprivation: in males and females (left panel) and in individuals aged ≤50 compared to >50 (right panel). PYAR, person years at risk

For peer review only

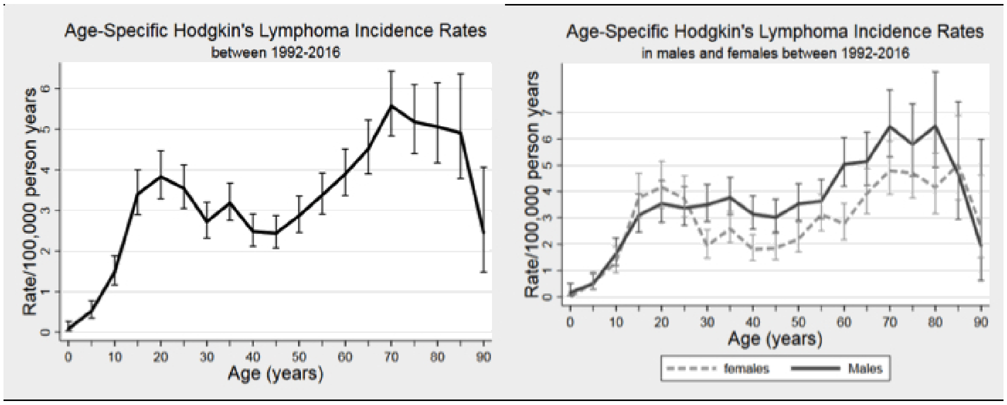

Figure 1: Age-specific Hodgkin's Lymphoma incidence in the study population (cohort of UK population): overall (left panel) and by sex (right panel), with 95% confidence interval bars

59x23mm (300 x 300 DPI)

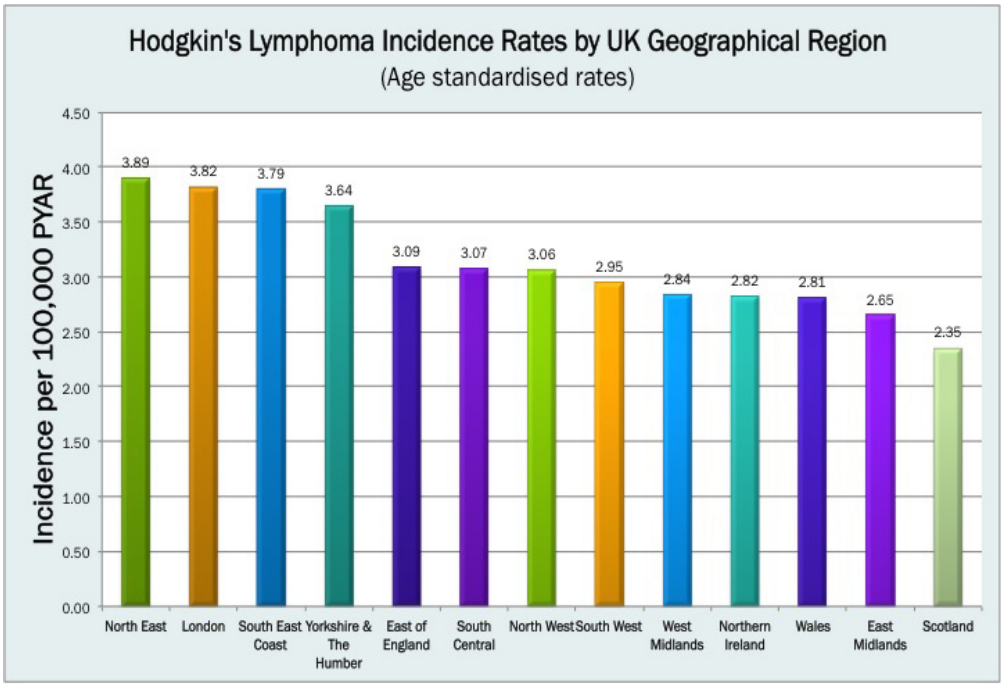

Figure 2: Age standardised Hodgkin's Lymphoma incidence in the study population (cohort of UK population) by region. PYAR, person years at risk

60x41mm (300 x 300 DPI)

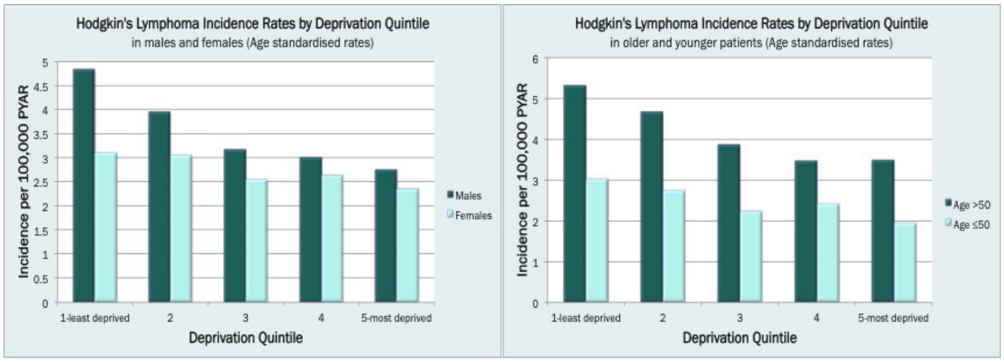

Figure 3: Age standardised Hodgkin's Lymphoma incidence in the study population (cohort of UK population) by deprivation: in males and females (left panel) and in individuals aged ≤50 compared to >50 (right panel). PYAR, person years at risk

72x25mm (300 x 300 DPI)

Supplementary Tables:

Supplementary Table 1: Read codes for Hodgkin's Lymphoma

B61..00	Hodgkin's disease
B61..11	Hodgkin lymphoma
B610.00	Hodgkin's paragranuloma
B610100	Hodgkin's paragranuloma of lymph nodes of head, face, neck
B610300	Hodgkin's paragranuloma of intra-abdominal lymph nodes
B611.00	Hodgkin's granuloma
B611100	Hodgkin's granuloma of lymph nodes of head, face and neck
B612.00	Hodgkin's sarcoma
B612400	Hodgkin's sarcoma of lymph nodes of axilla and upper limb
B613.00	Hodgkin's disease, lymphocytic-histiocytic predominance
B613000	Hodgkin's, lymphocytic-histiocytic predominance unspec site
B613100	Hodgkin's, lymphocytic-histiocytic pred of head, face, neck
B613200	Hodgkin's, lymphocytic-histiocytic pred intrathoracic nodes
B613300	Hodgkin's, lymphocytic-histiocytic pred intra-abdominal node
B613500	Hodgkin's, lymphocytic-histiocytic pred inguinal and leg
B613600	Hodgkin's, lymphocytic-histiocytic pred intrapelvic nodes
B613700	Hodgkin's, lymphocytic-histiocytic predominance of spleen
B613800	Hodgkin's, lymphocytic-histiocytic pred of multiple sites
B613z00	Hodgkin's, lymphocytic-histiocytic predominance NOS
B614.00	Hodgkin's disease, nodular sclerosis
B614000	Hodgkin's disease, nodular sclerosis of unspecified site
B614100	Hodgkin's nodular sclerosis of head, face and neck
B614200	Hodgkin's nodular sclerosis of intrathoracic lymph nodes
B614300	Hodgkin's nodular sclerosis of intra-abdominal lymph nodes
B614400	Hodgkin's nodular sclerosis of lymph nodes of axilla and arm
B614700	Hodgkin's disease, nodular sclerosis of spleen

For peer review only

B614800	Hodgkin's nodular sclerosis of lymph nodes of multiple sites
B614z00	Hodgkin's disease, nodular sclerosis NOS
B615.00	Hodgkin's disease, mixed cellularity
B615000	Hodgkin's disease, mixed cellularity of unspecified site
B615100	Hodgkin's mixed cellularity of lymph nodes head, face, neck
B615200	Hodgkin's mixed cellularity of intrathoracic lymph nodes
B615500	Hodgkin's mixed cellularity of lymph nodes inguinal and leg
B615z00	Hodgkin's disease, mixed cellularity NOS
B616.00	Hodgkin's disease, lymphocytic depletion
B616000	Hodgkin's lymphocytic depletion of unspecified site
B616400	Hodgkin's lymphocytic depletion lymph nodes axilla and arm
B616700	Hodgkin's disease, lymphocytic depletion of spleen
B616800	Hodgkin's lymphocytic depletion lymph nodes multiple sites
B616z00	Hodgkin's disease, lymphocytic depletion NOS
B617.00	Nodular lymphocyte predominant Hodgkin lymphoma
B618.00	Nodular sclerosis classical Hodgkin lymphoma
B619.00	Mixed cellularity classical Hodgkin lymphoma
B61B.00	Lymphocyte-rich classical Hodgkin lymphoma
B61C.00	Other classical Hodgkin lymphoma
B61z.00	Hodgkin's disease NOS
B61z.11	Hodgkin lymphoma NOS
B61z000	Hodgkin's disease NOS, unspecified site
B61z100	Hodgkin's disease NOS of lymph nodes of head, face and neck
B61z200	Hodgkin's disease NOS of intrathoracic lymph nodes
B61z300	Hodgkin's disease NOS of intra-abdominal lymph nodes
B61z400	Hodgkin's disease NOS of lymph nodes of axilla and arm
B61z500	Hodgkin's disease NOS of lymph nodes inguinal region and leg
B61z700	Hodgkin's disease NOS of spleen
B61z800	Hodgkin's disease NOS of lymph nodes of multiple sites

For peer review only

B61zz00	Hodgkin's disease NOS
BBj..00	[M]Hodgkin's disease
BBj0.00	[M]Hodgkin's disease NOS
BBj1.00	[M]Hodgkin's disease, lymphocytic predominance
BBj1000	[M]Hodgkin,s disease, lymphocytic predominance, diffuse
BBj1100	[M]Hodgkin,s disease, lymphocytic predominance, nodular
BBj2.00	[M]Hodgkin's disease, mixed cellularity
BBj4.00	[M]Hodgkin's disease,lymphocytic depletion,diffuse fibrosis
BBj6.00	[M]Hodgkin's disease, nodular sclerosis NOS
BBj6000	[M]Hodgkin,s disease, nodular sclerosis, lymphocytic predom
BBj6100	[M]Hodgkin,s disease, nodular sclerosis, mixed cellularity
BBj6200	[M]Hodgkin,s disease, nodular sclerosis, lymphocytic deplet
BBj7.00	[M]Hodgkin's disease, nodular sclerosis, cellular phase
BBj9.00	[M]Hodgkin's granuloma
BBjz.00	[M]Hodgkin's disease NOS
ByuD000	[X]Other Hodgkin's disease
ZV10711	[V]Personal history of Hodgkin's disease

Supplementary Table 2: ICD10 codes for Hodgkin's Lymphoma

Code	Term
C81	Hodgkin lymphoma
C81.0	Nodular lymphocyte predominant Hodgkin lymphoma
C81.1	Nodular sclerosis (classical) Hodgkin lymphoma
C81.2	Mixed cellularity (classical) Hodgkin lymphoma
C81.3	Lymphocyte depleted (classical) Hodgkin lymphoma
C81.4	Lymphocyte-rich (classical) Hodgkin lymphoma
C81.7	Other (classical) Hodgkin lymphoma

For peer review only

C81.9	Hodgkin lymphoma unspecified

For peer review only

Supplementary Table 3: Age-specific incidence rates of Hodgkin's Lymphoma by sex. CI, confidence interval; ASR, age standardised rate

Age group (years)	Incidence Rate Overall	95% CI	Incidence Rate Males	95% CI	Incidence Rate Females	95% CI	Incidence Rate Ratio (males / females)
0-4	0.09	0.03–0.27	0.17	0.05–0.52	0.00	–	
5-9	0.52	0.35–0.77	0.50	0.29–0.89	0.54	0.31–0.95	0.94
10-14	1.48	1.17–1.88	1.63	1.19–2.23	1.32	0.92–1.90	1.23
15-19	3.40	2.89–4.00	3.09	2.45–3.90	3.75	3.00–4.70	0.82
20-24	3.83	3.29–4.47	3.53	2.83–4.41	4.16	3.36–5.16	0.85
25-29	3.55	3.05–4.12	3.37	2.71–4.19	3.73	3.02–4.59	0.90
30-34	2.72	2.31–3.20	3.49	2.85–4.27	1.94	1.48–2.55	1.80
35-39	3.18	2.76–3.67	3.76	3.13–4.53	2.59	2.06–3.24	1.46
40-44	2.48	2.11–2.91	3.14	2.57–3.83	1.80	1.38–2.35	1.74
45-49	2.44	2.07–2.87	3.01	2.45–3.70	1.85	1.41–2.41	1.63
50-54	2.87	2.46–3.35	3.53	2.89–4.29	2.20	1.70–2.83	1.61
55-59	3.38	2.91–3.92	3.63	2.96–4.44	3.12	2.50–3.89	1.16
60-64	3.90	3.37–4.51	5.04	4.20–6.05	2.77	2.17–3.54	1.82
65-69	4.51	3.90–5.22	5.15	4.23–6.26	3.92	3.15–4.87	1.31
70-74	5.58	4.83–6.43	6.47	5.33–7.86	4.80	3.89–5.92	1.35
75-79	5.18	4.40–6.10	5.80	4.58–7.32	4.71	3.75–5.91	1.23
80-84	5.06	4.17–6.15	6.49	4.93–8.54	4.15	3.15–5.46	1.56
85-89	4.91	3.79–6.37	4.67	2.94–7.41	5.03	3.68–6.89	0.93
90+	2.45	1.48–4.07	1.93	0.62–5.99	2.63	1.49–4.63	0.73
**ASR**	**3.10**	**2.98–3.22**	**3.51**	**3.32–3.70**	**2.72**	**2.56–2.88**	

Supplementary Table 4: Crude and age-standardised Hodgkin's Lymphoma incidence rates in the UK by sex, age group, deprivation and geographical region. PYAR, person years at risk; ASR, age standardised rate; CI, confidence interval; IMD, index of multiple deprivation; yrs, age group in years

	Cases	PYAR	Incidence Rate per 100,000 PYAR	ASR per 100,000 PYAR	95%CI
Male	1331	39,039,332	3.41	3.51	3.32–3.70
Female	1071	39,529,340	2.71	2.72	2.56–2.88
IMD 1 (least deprived)	572	14,880,179	3.84	3.92	3.60–4.24
IMD 2	500	14,627,384	3.42	3.49	3.18–3.79
IMD 3	456	15,954,225	2.86	2.86	2.60–3.13
IMD 4	472	16,850,996	2.80	2.82	2.57–3.07
IMD 5 (most deprived)	402	16,256,652	2.47	2.55	2.30–2.80
North East England	53	1,419,931	3.73	3.89	2.84–4.94
Yorkshire/Humber	105	2,938,253	3.57	3.64	2.94–4.33
London	299	8,287,047	3.61	3.82	3.38–4.25
South East Coast	284	7,564,683	3.75	3.79	3.35–4.23
East of England	213	7,012,254	3.04	3.09	2.68–3.51
North West England	275	9,159,626	3.00	3.06	2.70–3.42
South West England	197	6,598,934	2.99	2.95	2.54–3.36
West Midlands	198	7,054,011	2.81	2.84	2.44–3.24
Wales	202	7,155,514	2.82	2.81	2.42–3.20
South Central England	256	8,482,798	3.02	3.07	2.70–3.45
East Midlands	80	3,042,062	2.63	2.65	2.07–3.23
Northern Ireland	70	2,597,902	2.69	2.82	2.16–3.48
Scotland	170	7,256,423	2.34	2.35	2.00–2.70
0-4yrs	3	3,507,576	0.09		
5-9yrs	24	4,620,605	0.52		
10-14yrs	68	4,590,771	1.48		
15-19yrs	147	4,321,501	3.40		
20-24yrs	162	4,226,413	3.83		
25-29yrs	170	4,794,244	3.55		
30-34yrs	147	5,402,587	2.72		
35-39yrs	187	5,877,364	3.18		
40-44yrs	151	6,089,433	2.48		
45-49yrs	145	5,943,893	2.44		
50-54yrs	159	5,541,281	2.87		
55-59yrs	172	5,094,300	3.38		
60-64yrs	179	4,592,514	3.90		
65-69yrs	181	4,009,358	4.51		
70-74yrs	189	3,390,115	5.58		
75-79yrs	144	2,779,666	5.18		
80-84yrs	102	2,015,256	5.06		
85-89yrs	57	1,160,678	4.91		
90+yrs	15	611,886	2.45		
**Overall ASR**				**3.10**	**2.98–3.22**

Reporting checklist for cohort study.

Based on the STROBE cohort guidelines.

Instructions to authors

Complete this checklist by entering the page numbers from your manuscript where readers will find each of the items listed below.

Your article may not currently address all the items on the checklist. Please modify your text to include the missing information. If you are certain that an item does not apply, please write "n/a" and provide a short explanation.

Upload your completed checklist as an extra file when you submit to a journal.

In your methods section, say that you used the STROBE cohort reporting guidelines, and cite them as:

von Elm E, Altman DG, Egger M, Pocock SJ, Gotzsche PC, Vandenbroucke JP. The Strengthening the Reporting of Observational Studies in Epidemiology (STROBE) Statement: guidelines for reporting observational studies.

		Reporting Item	Page Number
Title	#1a	Indicate the study's design with a commonly used term in the title or the abstract	1
Abstract	#1b	Provide in the abstract an informative and balanced summary of what was done and what was found	3
Background / rationale	#2	Explain the scientific background and rationale for the investigation being reported	6
Objectives	#3	State specific objectives, including any prespecified hypotheses	6
Study design	#4	Present key elements of study design early in the paper	7, 8
Setting	#5	Describe the setting, locations, and relevant dates, including periods of recruitment, exposure, follow-up, and data collection	7, 8
Eligibility criteria	#6a	Give the eligibility criteria, and the sources and methods of selection of participants. Describe methods of follow-up.	7, 8
	#6b	For matched studies, give matching criteria and number of exposed and	n/a

For peer review only

		unexposed	
Variables	#7	Clearly define all outcomes, exposures, predictors, potential confounders, and effect modifiers. Give diagnostic criteria, if applicable	8, 9
Data sources / measurement	#8	For each variable of interest give sources of data and details of methods of assessment (measurement). Describe comparability of assessment methods if there is more than one group. Give information separately for for exposed and unexposed groups if applicable.	7, 8
Bias	#9	Describe any efforts to address potential sources of bias	7, 8, 9
Study size	#10	Explain how the study size was arrived at	7, 8
Quantitative variables	#11	Explain how quantitative variables were handled in the analyses. If applicable, describe which groupings were chosen, and why	8
Statistical methods	#12a	Describe all statistical methods, including those used to control for confounding	8, 9
	#12b	Describe any methods used to examine subgroups and interactions	9
	#12c	Explain how missing data were addressed	n/a
	#12d	If applicable, explain how loss to follow-up was addressed	7, 8
	#12e	Describe any sensitivity analyses	n/a
Participants	#13a	Report numbers of individuals at each stage of study—eg numbers potentially eligible, examined for eligibility, confirmed eligible, included in the study, completing follow-up, and analysed. Give information separately for for exposed and unexposed groups if applicable.	10
	#13b	Give reasons for non-participation at each stage	n/a
	#13c	Consider use of a flow diagram	n/a
Descriptive data	#14a	Give characteristics of study participants (eg demographic, clinical, social) and information on exposures and potential confounders. Give information separately for exposed and unexposed groups if applicable.	10
	#14b	Indicate number of participants with missing data for each variable of interest	n/a
	#14c	Summarise follow-up time (eg, average and total amount)	10

For peer review only

Outcome data	#15	Report numbers of outcome events or summary measures over time. Give information separately for exposed and unexposed groups if applicable.	10
Main results	#16a	Give unadjusted estimates and, if applicable, confounder-adjusted estimates and their precision (eg, 95% confidence interval). Make clear which confounders were adjusted for and why they were included	10, 11
	#16b	Report category boundaries when continuous variables were categorized	10, 11
	#16c	If relevant, consider translating estimates of relative risk into absolute risk for a meaningful time period	n/a
Other analyses	#17	Report other analyses done—e.g., analyses of subgroups and interactions, and sensitivity analyses	11
Key results	#18	Summarise key results with reference to study objectives	12
Limitations	#19	Discuss limitations of the study, taking into account sources of potential bias or imprecision. Discuss both direction and magnitude of any potential bias.	14
Interpretation	#20	Give a cautious overall interpretation considering objectives, limitations, multiplicity of analyses, results from similar studies, and other relevant evidence.	15, 16
Generalisability	#21	Discuss the generalisability (external validity) of the study results	13, 14
Funding	#22	Give the source of funding and the role of the funders for the present study and, if applicable, for the original study on which the present article is based	1, 2

The STROBE checklist is distributed under the terms of the Creative Commons Attribution License CC-BY.

This checklist was completed on 17. January 2019 using https://www.goodreports.org/, a tool made by the EQUATOR Network in collaboration with Penelope.ai

For peer review only
